# EM-mosaic detects mosaic point mutations that contribute to congenital heart disease

**DOI:** 10.1186/s13073-020-00738-1

**Published:** 2020-04-29

**Authors:** Alexander Hsieh, Sarah U. Morton, Jon A. L. Willcox, Joshua M. Gorham, Angela C. Tai, Hongjian Qi, Steven DePalma, David McKean, Emily Griffin, Kathryn B. Manheimer, Daniel Bernstein, Richard W. Kim, Jane W. Newburger, George A. Porter, Deepak Srivastava, Martin Tristani-Firouzi, Martina Brueckner, Richard P. Lifton, Elizabeth Goldmuntz, Bruce D. Gelb, Wendy K. Chung, Christine E. Seidman, J. G. Seidman, Yufeng Shen

**Affiliations:** 1 Columbia University Medical Center, 1130 St Nicholas Ave, New York, NY 10032 USA; 2 Boston Children’s Hospital, Boston, MA USA; 3 Harvard Medical School, Boston, MA USA; 4 Icahn School of Medicine at Mount Sinai, New York, NY USA; 5 Stanford University, Palo Alto, CA USA; 6 Children’s Hospital Los Angeles, Los Angeles, CA USA; 7 University of Rochester Medical Center, Rochester, NY USA; 8 Gladstone Institutes and University of California San Francisco, San Francisco, CA USA; 9 University of Utah School of Medicine, Salt Lake City, UT USA; 10 Yale University School of Medicine, New Haven, CT USA; 11 Rockefeller University, New York, NY USA; 12 Children’s Hospital of Philadelphia, Philadelphia, PA USA; 13 Brigham and Women’s Hospital, Boston, MA USA; 14 Howard Hughes Medical Institute, Harvard University, Boston, MA USA

**Keywords:** Mosaic, Somatic, Congenital heart disease, Exome sequencing

## Abstract

**Background:**

The contribution of somatic mosaicism, or genetic mutations arising after oocyte fertilization, to congenital heart disease (CHD) is not well understood. Further, the relationship between mosaicism in blood and cardiovascular tissue has not been determined.

**Methods:**

We developed a new computational method, EM-mosaic (Expectation-Maximization-based detection of mosaicism), to analyze mosaicism in exome sequences derived primarily from blood DNA of 2530 CHD proband-parent trios. To optimize this method, we measured mosaic detection power as a function of sequencing depth. In parallel, we analyzed our cohort using MosaicHunter, a Bayesian genotyping algorithm-based mosaic detection tool, and compared the two methods. The accuracy of these mosaic variant detection algorithms was assessed using an independent resequencing method. We then applied both methods to detect mosaicism in cardiac tissue-derived exome sequences of 66 participants for which matched blood and heart tissue was available.

**Results:**

EM-mosaic detected 326 mosaic mutations in blood and/or cardiac tissue DNA. Of the 309 detected in blood DNA, 85/97 (88%) tested were independently confirmed, while 7/17 (41%) candidates of 17 detected in cardiac tissue were confirmed. MosaicHunter detected an additional 64 mosaics, of which 23/46 (50%) among 58 candidates from blood and 4/6 (67%) of 6 candidates from cardiac tissue confirmed. Twenty-five mosaic variants altered CHD-risk genes, affecting 1% of our cohort. Of these 25, 22/22 candidates tested were confirmed. Variants predicted as damaging had higher variant allele fraction than benign variants, suggesting a role in CHD. The estimated true frequency of mosaic variants above 10% mosaicism was 0.14/person in blood and 0.21/person in cardiac tissue. Analysis of 66 individuals with matched cardiac tissue available revealed both tissue-specific and shared mosaicism, with shared mosaics generally having higher allele fraction.

**Conclusions:**

We estimate that ~ 1% of CHD probands have a mosaic variant detectable in blood that could contribute to cardiac malformations, particularly those damaging variants with relatively higher allele fraction. Although blood is a readily available DNA source, cardiac tissues analyzed contributed ~ 5% of somatic mosaic variants identified, indicating the value of tissue mosaicism analyses.

**Electronic supplementary material:**

The online version of this article (10.1186/s13073-020-00738-1) contains supplementary material, which is available to authorized users.

## Background

Mosaicism results from somatic mutations that arise post-zygotically in an early embryonic cell, resulting in two or more cell populations with distinct genotypes in the developing embryo [4]. The developmental status of the early embryonic cell at the time of mutagenesis determines the proportion of variant-carrying cells and the tissue distribution of these cells in the post-natal child [1]. While germline variants have a variant allele frequency (VAF) of 0.5, somatic mosaic variants have a significantly lower VAF.

Post-zygotic mosaic mutations have been implicated in several diseases including non-malignant developmental disorders such as overgrowth syndromes [47, 55, 64], structural brain malformations [41, 49, 64, 69], epilepsy [76], and autism spectrum disorder [16, 23, 45, 54]. Recent analyses also identified mosaic variants in a cohort of patients with congenital heart disease (CHD) [57], but the prevalence of these was far less than germline variants (CHD) [34, 42, 86, 87].

Assessment of the frequency of mosaicism in human disease is confounded by technical issues, including differences in sequencing depth, DNA sources, and variant assessment pipelines. Low levels of mosaicism can escape the detection threshold of traditional sequencing methods with standard read depths, while post-zygotic mutations with a higher percentage of affected cells are difficult to discriminate from germline de novo mutations [1]. All of these issues can lead to substantially different conclusions. For example, analyses of mosaicism in autism spectrum disorder was recently assessed from whole exome sequence (WES) data from whole blood DNA from 2506 families (proband, parents and unaffected sibling; trios and quads) in the Simons Simplex Collection (SSC) [21]. The primary sequence data were analyzed by three groups; one that identified a protein-coding somatic mosaic variant rate of 0.074 per individual [23], another that found a mosaic rate of 0.059 per individual [54], and a third group that reported a mosaic rate of 0.125 per individual [45]. This disparity both highlights algorithm-specific differences and suggests the need for a more systematic mosaic mutation detection method that accounts for dataset-specific confounding factors.

By contrast, analyses of affected tissues can improve the sensitivity and specificity of detection of somatic mosaicism. In cancer, methods to detect these events, such as MuTect [7], compare tumor and benign tissues from the same patient. Mosaicism has also been demonstrated from the analyses of unpaired samples with cancer and other pathologies [37, 73, 77] by the demonstration of variants in affected tissues that are absent from blood-derived DNA [59, 78]. With access to cardiac tissues from patients with CHD obtained during surgical repair, we hypothesized that analyses of mosaicism in cardiac tissue might improve insights into the causes of this common congenital anomaly. As many cardiomyocyte lineages share a mesodermic origin with blood cells but exit the cell cycle during embryogenesis, we also sought to determine if mosaicism in the heart exhibited distinct patterns of mosaicism with regard to variant frequency and allele fractions.

In this study, we developed a computational method, EM-mosaic (Expectation-Maximization-based detection of Mosaicism) [35], to detect mosaic single-nucleotide variants (SNVs) using WES of proband and parent DNA. To optimize this method, we measured mosaic detection power as a function of sequencing depth. We applied both EM-mosaic and MosaicHunter [37] to investigate mosaicism in 2530 CHD proband-parent trios from the Pediatric Cardiac Genomics Consortium (PCGC) [42], using exome sequences derived from blood-derived DNA, and compared the two methods. We detected predicted deleterious mosaic mutations in genes involved in known biological processes relevant to CHD or developmental disorders in 1% of probands. The accuracy of these mosaic variant detection algorithms was assessed using an independent resequencing method. We found that among high-confidence mosaic mutations in CHD-relevant genes, likely damaging variants tended to have higher VAF than likely benign variants.

In parallel, we assessed mosaicism by EM-mosaic and MosaicHunter in 70 discarded tissues from several heart regions obtained from 66 probands who underwent cardiac surgical repairs. While VAF varied significantly (> 3 fold) between blood and cardiovascular tissue at about 60% of sites, in general mosaic variants with high (> 15%) VAF were more likely shared between blood and cardiac tissue than variants with lower VAF.

## Methods

### Samples and sequencing data

We analyzed WES data from 2530 congenital heart disease (CHD) proband-parents trio families who were recruited as part of the Pediatric Cardiac Genomics Consortium (PCGC) study [34, 42]. Genomic DNA from venous blood or saliva was captured using Nimblegen v.2 exome capture reagent (Roche) or Nimblegen SeqCap EZ MedExome Target Enrichment Kit (Roche) followed by Illumina DNA sequencing (paired-end, 2x75bp) [42, 86]. Of 2530 participant DNA samples, 2453 were from blood and 77 were from saliva. Genomic DNA from 70 surgically discarded cardiovascular tissue samples (2-10 mg) was isolated using DNeasy Blood & Tissue Kit (QIAgen), then captured using xGen Exome Research Panel v1.0 reagent (IDT) followed by Illumina DNA sequencing (paired-end, 2 × 75 bp). Sequence reads were mapped to the hg19 human reference genome with BWA-MEM [51], and BAM files were further processed following GATK Best Practices [81], which included duplication marking, indel realignment, and base quality recalibration steps. Blood and saliva samples had sample average depth 60× and cardiovascular tissue samples had sample average depth 160×. A summary of germline and mosaic variants called from blood and saliva DNA can be found in Additional file 1: Table S15.

### De novo variant calling and annotation

We processed our sample BAMs and called variants on a per-trio basis using SAMtools (v1.3.1-42) and BCFtools (v1.3.1-174) [52]. Pileups were generated using samtools “*mpileup*” command with mapQ 20 and baseQ 13 to minimize the effect of poorly mapped reads on variant allele fraction, followed by bcftools “*call*” using a cutoff of 1.1 for the posterior probability of the homozygous reference genotype parameter (-p) to capture additional sites with variant allele fraction suggestive of post-zygotic origin that would otherwise be excluded under the default threshold of 0.01. To identify de novo mutations from trio VCF files, we selected sites with (i) a minimum of 6 reads supporting the alternate allele in the proband and (ii) for both parents, a minimum depth of 10 reads and 0 alternate allele read support. Variants were then annotated using ANNOVAR (v2017-07-17) [83] to include information from refGene [63], gnomAD (March 2017) [44], 1000 Genomes (August 2015) [2], ExAC [43], genomicSuperDups [32], CADD (v1.3) [68], COSMIC (v70) [79], and dbSNP (v147) [56] databases, as well as pathogenicity predictions from a variety of established methods included as part of the dbNSFP (v3.0a) database or generated in-house (MCAP, REVEL, MVP, MPC). We used REVEL [38] to evaluate missense variant functional consequence, using the recommended threshold of 0.5 corresponding to sensitivity of 0.754 and specificity of 0.891. We used spliceAI [39] to predict the variant functional impact on splicing using the delta score thresholds of 0.2 for likely pathogenic (high recall), 0.5 for pathogenic (recommended), and 0.8 for pathogenic (high precision). We considered sites predicted to be loss of function (LoF) (stopgain, stoploss, frameshift indels, splice-site), deleterious missense (Dmis; nonsynonymous SNV with REVEL> 0.5), or splice-damaging (benign missense or synonymous SNV with delta score > 0.5) to be damaging and likely disease causing. We considered sites predicted to be synonymous (delta score ≤ 0.5) or benign missense (Bmis; nonsynonymous SNV with REVEL ≤ 0.5 and delta score ≤ 0.5) to be non-damaging.

### Pre-processing and QC

**Fig. 1:**
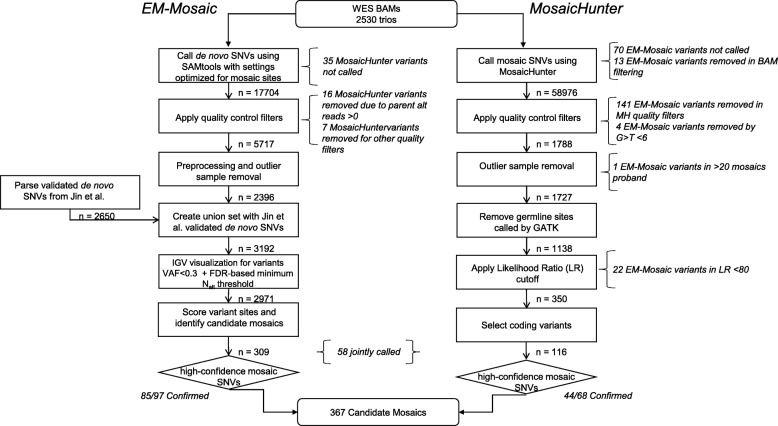
Mosaic detection pipeline flowchart. Summary of approach for detecting mosaic variants in our cohort of *n* = 2530 CHD proband-parent trios. EM-mosaic flowchart (left). We first processed our SAMtools de novo calls using our upstream filters (*n* = 2396 sites passing all filters). We then applied the same upstream filters to the published dnSNVs from Jin et al. (*n* = 2650 sites passing all filters) before finally taking the union of these two call sets (*n* = 3192). High-confidence mosaics (*n* = 309) were defined as mosaics passing IGV inspection and having posterior odds > 10. Italicized text indicates which filters removed candidate mosaic variants called by MosaicHunter but not by EM-mosaic. MosaicHunter workflow (right). Quality control filters excluded any sites that were (1) present in ExAC (2) G>T with *N*_alt_ < 10 (3) parent *N*_alt_ > 2. Outliers were defined as probands carrying more than 20 mosaics, or non-unique sites. We also removed sites called as germline by GATK Haplotype Caller. High-confidence mosaics (*n* = 116) were defined as having a likelihood ratio > 80 and affecting coding regions excluding MUC/HLA genes. Italicized text indicates which filters removed variants called by EM-mosaic but not by MosaicHunter

To reduce the number of low VAF technical artifacts introduced by our variant calling approach, we preprocessed our variants using a variety of filters (Fig. 1). We first excluded indels from further analysis, as their downstream model parameter estimates were less stable than those of SNVs. We then filtered our variant call set for rare heterozygous coding mutations (minor allele frequency (MAF) ≤ 10^− 4^ across all populations represented in gnomAD and ExAC databases). To account for regions in the reference genome that are likely to affect read depth estimates, we removed variant sites found in regions of non-unique mappability (score < 1; 300 bp), likely segmental duplication (score > 0.95), and known low-complexity [53]. We then excluded sites located in MUC and HLA genes and imposed a maximum variant read depth threshold of 500. We used SAMtools PV4 to exclude sites with evidence of technical issues using a cutoff of 1e−3 for baseQ bias and tail distance bias and a cutoff of 1e−6 for mapQ bias. To account for potential strand bias, we used an in-house script to flag sites that have either (1) 0 alternate allele read support on either the forward or reverse strand or (2) *P* < 1e−3 and (odds ratio (OR) < 0.33 or OR > 3) when applying a two-sided Fisher’s exact test to compare proportions of reference and alternate allele read counts on the forward and reverse strands. We also excluded sites with cohort frequency > 1%, as well as sites belonging to outlier samples (with abnormally high de novo SNV (dnSNV) counts, cutoff = 8) and variant clusters (defined as sites with neighboring SNVs within 10 bp). Finally, we applied an false discovery rate (FDR)-based minimum *N*_alt_ filtering step (Additional file 2: Figure S5) to control for false positives caused purely by sequencing errors.

### IGV visualization of low allele fraction de novo SNVs

To reduce the impact of technical artifacts on model parameter estimation, we manually inspected de novo SNVs with VAF < 0.3 (*n* = 558) using Integrative Genomics Viewer (v2.3.97) [70] to visualize the local read pileup at each variant across all members of a given trio family. We focused on the allele fraction range 0.0–0.3 since this range is enriched for technical artifacts that could potentially impact downstream parameter estimation. Sites were filtered out if (1) there are inconsistent mismatches in the reads supporting the mosaic allele, (2) the site overlaps or is adjacent to an indel, (3) the site has low MAPQ or is not primary alignment, (4) there is evidence of technical bias (strand, read position, tail distance), or (5) the site is mainly supported by soft-clipped reads.

### Expectation-maximization to estimate prior mosaic fraction and control FDR

Current estimates for the fraction of de novo events occurring post-zygotically are unstable due to differences in study factors such as variant calling methods, average sequencing depth, and paternal ages. In order to use this fraction as a prior probability in our posterior odds and false discovery calculations, we reason that this value must be estimated from the data itself. We used an expectation-maximization algorithm to jointly estimate the fraction of mosaics among apparent de novo mutations and to calculate a per-site likelihood ratio score. This initial mosaic fraction estimate gives us a prior probability of mosaicism, independent of sequencing depth or variant caller, and allows us to calculate for each variant the posterior odds that a given site is mosaic rather than germline. To control for false discovery among our predicted mosaic candidates, we chose a posterior odds threshold of 10 to restrict FDR to 9.1%.

### Mosaic mutation detection model

To distinguish variant sites that show evidence of mosaicism from germline heterozygous sites, we modeled the number of reads supporting the variant allele (*N*_alt_) as a function of the total site depth (*N*). In the typical case, *N*_alt_ follows a binomial model with parameters *N* = site depth and *P* = mean VAF. However, we observed notable overdispersion in the distribution of variant allele fraction compared to the expectations under this binomial model (Additional file 2: Figure S4). To account for this overdispersion, we instead modeled *N*_alt_ using a beta-binomial distribution [33, 66]. We estimated an overdispersion parameter *θ* for our model as follows: for site depth values *N* in the range 1 to 500, we (1) bin variants by identifying all sites with depth *N*, (2) calculate a maximum-likelihood estimate *θ* value using *N* and all *N*_alt_ values for variants in a given bin, and (3) estimate a global *θ* value by taking the average of *θ* values across all bins, weighted by the number of variants in each bin. We then used *θ* in our expectation-maximization approach to jointly estimate prior mosaic fraction and to calculate per-site likelihood ratios.

To calculate the posterior odds that a given variant arose post-zygotically, we first calculated a likelihood ratio (LR) of two models: M_0_: germline heterozygous variant, and M_1_: mosaic variant. Under our null model M_0_, we calculated the probability of observing *N*_alt_ from a beta-binomial distribution with site depth *N*, observed mean germline VAF *P*, and overdispersion parameter *θ*. Under our alternate model M_1_, we calculated the probability of observing *N*_alt_ from a beta-binomial distribution with site depth *N*, observed site VAF *P* = *N*_alt_*/N*, and overdispersion parameter *θ*. Finally, for each variant, we calculated LR by using the ratio of probabilities under each model and posterior odds by multiplying LR by our E-M estimated prior mosaic fraction estimate. We defined mosaic sites as those with posterior odds greater than 10 (corresponding to 9.1% FDR). We used posterior odds in this context to be able to control for false discovery, but we output similarly valid *P* value and likelihood ratio scores for each de novo SNV.

### Mutation confirmation by MiSeq amplicon sequencing

Chromosome coordinates were expanded 500 bp upstream and downstream of the candidate mosaic variants in the UCSC Genome Browser. Primer 3 Plus software was used to design forward and reverse primers to generate 150–300-bp amplimers containing the candidate site. PCR reactions consisting of genomic DNA, primers, and Phusion polymerase were amplified by thermal cycling and purified with AMPure XP beads. The purified PCR product was quantified, and 0.5–1.0 ng of product was used to construct Nextera XT libraries according to the protocol published by Illumina. Libraries were purified using AMPure XP beads, and final libraries were quantified and pooled to undergo sequencing through Illumina MiSeq.

We experimentally tested for the presence our predicted post-zygotic sites in the original blood DNA and cardiovascular tissue DNA samples using Illumina MiSeq Amplicon sequencing. The Amplicon Deep Sequencing workflow, optimized for the detection of somatic mutations in tumor samples, offers ultra-high sequencing depth (> 1000×) that gives us the resolution to confirm low VAF variants, to accurately estimate site VAF, and to distinguish true variant calls from technical artifacts. Mosaic candidates were considered validated if the variant allele matched the MiSeq call and both the mosaic VAF and MiSeq VAF indicated post-zygotic origin (VAF < 0.45).

Mosaic candidates were selected for confirmation on the basis of VAF, plausible involvement in CHD (based on predicted pathogenicity and HHE status), and detection method (Additional file 1: Table S11; Additional file 1: Table S12). We sampled mosaics from both ends of the VAF spectrum to evaluate our ability to distinguish high VAF mosaics (VAF > 0.2; *n* = 29) from germline variants and to distinguish low VAF mosaics (VAF < =0.1; *n* = 52) from technical artifacts. Confirmation rate across different VAF bins is shown in Additional file 2: Figure S12. We also selected for confirmation mosaics detected uniquely by either EM-mosaic or MosaicHunter, for the sake of method comparison (Table 1).

**Table 1:** Mosaic variant detection by EM-Mosaic and MosaicHunter and validated by PCR product sequencing

	**Union**	**Shared**	**Unique**	
			**EM-Mosaic**	**MosaicHunter**
High-confidence mosaic variants*	**332**	**57**	**240**	**35**
**Mosaic candidates**	367	58	251	58
**Mosaic candidate VAF mean (SD)**	0.13 (0.06)	0.12 (0.05)	0.13 (0.06)	0.10 (0.05)
**MiSeq confirmation**				
Total tested	143	22	75	46
Mosaic	108	21	64	23
Germline	3	0	3	0
No variant	32	1	8	23
**Validation rate**	76%	95%	85%	50%

*Mosaic variants detected from blood DNA of 2530 CHD probands, after excluding sites failing MiSeq confirmation

To examine a potential source of bias in our candidate selection process, we compared the posterior odds distribution of selected candidate mosaics (*n* = 97) against those not chosen (*n* = 212). We found that our tested candidates had lower posterior odds than untested mosaics (mean_tested_ = 5.382, mean_untested_ = 7.050, log_10_-scale; Mann-Whitney *U P* = 0.002) (Figure 13), suggesting that our validation rate is not buoyed by testing variants with the strongest evidence of mosaicism. For method development purposes, we intentionally focused on mosaics with lower posterior odds as these fall in the VAF range for which it is most difficult to distinguish germline from mosaic.

### Investigating the relationship between VAF and pathogenicity

We hypothesized that mosaic contribution to disease is positively correlated with cellular percentage and by extension mutational timing. Here, we used variant allele fraction as a proxy for cellular percentage. We grouped mosaics into likely damaging and likely benign and compared the distribution of allele fraction in CHD-related genes. We defined likely damaging variants as (a) likely gene-disrupting (LOF) variants (including premature stopgain, frameshifting, and variants located in canonical splice sites), (b) missense variants predicted to be damaging by REVEL [38] (with score ≥ 0.5), or (c) missense variants and synonymous predicted to be splice-damaging by spliceAI (with score > 0.5). One of the main findings from previous CHD studies is that damaging de novo variants in genes highly expressed in the developing heart (“HHE”, ranked in the top 25% by cardiac expression data in mouse at E14.5 [34, 86]) contribute to non-isolated CHD cases that have additional congenital anomalies or neurodevelopmental disorders. Therefore, we considered the union of HHE genes and known candidate CHD genes [42] as CHD-related genes (*n* = 4558). For mosaics in CHD-related genes and for mosaics in other genes, we used a Mann-Whitney *U* test to compare the VAF distributions of likely damaging and likely benign groups.

### Estimated contribution of mosaicism to CHD

We identified likely disease-causing mosaic mutations on the basis of predicted pathogenicity and presence in genes involved in biological processes relevant to CHD or developmental disorders. Each mosaic mutation was annotated with gene-specific information, including heart expression percentile, probability of loss-of-function intolerance (pLI) score [50], whether dysregulation causes CHD in mice [20, 72], and gene function (NCBI RefSeq). We focused on HHE genes, genes with high pLI (pLI > 0.9), genes that cause CHD phenotypes in mice, and genes involved in key developmental processes such as Wnt, mTOR, and TGF-beta signaling pathways. Then, for each patient, we used the clinical phenotype to further prioritize mosaic mutations most likely contributing to that individual’s clinical features. Detailed mutation annotation and clinical phenotypes for the mosaic carriers described above can be found in Additional file 1: Table S10. We estimate the contribution of mosaicism to CHD as the percentage of individuals carrying likely disease-causing mosaic mutations among all individuals in our CHD cohort.

## Results

### High-accuracy detection of mosaic mutations in WES data using EM-mosaic

We analyzed whole exome sequence (WES) data from 2530 CHD proband-parent trios [34, 42] (Additional file 1: Table S1). Among this cohort, 1205 probands had CHD with neurodevelopmental disorders (NDD) and/or extracardiac manifestations (EM), 788 had isolated CHD at the time of enrollment, 539 had undetermined NDD status due to young neonatal age at the time of enrollment, and 9 subjects had incomplete data (Additional file 1: Table S2).

Previous WES analyses [42] identified 1742 germline de novo SNVs carried by 2005 CHD proband-parent trios, including 838 cases with NDD and/or EM, 516 isolated cases, 644 cases of unknown NDD status, and 7 with incomplete data. These de novo variants were identified using the Genome Analysis Toolkit (GATK) pipeline [14, 58] assuming a germline diploid model in which the expected VAF is 0.5. This model has limited sensitivity to detect mosaic mutations for which the fraction of alternative allele reads is significantly below 0.5, especially because de novo variants with VAF < 0.2 were excluded to reduce false discovery.

To efficiently capture mosaic variants with VAF < 0.4, we developed a new method (EM-mosaic) to detect mosaic variants in WES sequence of a proband and parents (trios). Potential mosaic variants were identified in WES sequence data using SAMtools *mpileup* [52] with settings designed to capture sites with VAF between 0.1–0.4 and merged with the variants found by the GATK pipeline [42] (Fig. 1) to create a union variant set. To reduce the elevated false positive rate inherent in low-VAF calls, we applied a set of empirical filters to remove likely technical artifacts due to sequencing errors associated with repetitive and/or low-complexity sequences. We then manually inspected de novo SNVs with VAF < 0.3 (*n* = 582) using IGV and filtered out an additional 188 likely false positives. After preprocessing, outlier removal, and an FDR-based minimum alternate allele read support (*N*_alt_) filter (Additional file 2: Figure S5), the remaining 2971 de novo SNVs were used as input to our mosaic detection model.

**Fig. 2:**
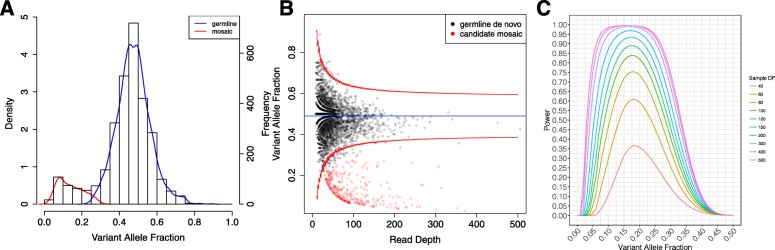
Mosaic detection by Expectation-Maximization. **a** Expectation-Maximization (EM) estimation to decompose the variant allele fraction (VAF) distribution of our input variants into mosaic and germline distributions. The EM-estimated prior mosaic fraction was 12.15% and the mean of the mosaic VAF distribution was 0.15. **b** Read depth vs. VAF distribution of individual variants. The blue line denotes mean VAF (0.49) and the red lines denote the 95% confidence interval under our Beta-Binomial model. Mosaic variants are defined as sites with posterior odds > 10, corresponding to a false discovery rate of 9.1%. Germline variants are represented in black and mosaic variants are represented in red. **c** Estimated mosaic detection power as a function of average sample depth for values between 40× and 500×

Among the 2971 de novo SNVs, this pipeline identified 309 sites as candidate mosaics based on posterior odds score (Fig. 2a, b; Additional file 1: Table S3), including 50 sites that were previously reported as germline de novo variants [42]. Among our 2530 participant DNA samples, 2453 were from blood and 77 were from saliva yielding 300 mosaic candidates and 9 mosaic candidates, respectively; a summary of germline and mosaic variants called from blood and saliva DNA can be found in Additional file 1: Table S15. We also did not find evidence of a relationship between proband age and mosaic rate (Additional file 2: Figure S9) or between parental age (paternal or maternal) and proband mosaic rate (Additional file 1: Table S16; Additional file 2: Figure S10; Additional file 2: S11). Among sites predicted to be germline, 86 sites were identified as having posterior odds below our chosen threshold of 10 but greater than 1 (Additional file 2: Figure S1), including a *ZEB2* variant with a posterior odds score of 4.7 that was previously confirmed via ddPCR [57]. Among these 86 variants, we estimate that 53 are likely mosaic and 33 are likely germline (Additional file 2: Figure S1B). We chose not to include these sites since there was insufficient evidence to confidently resolve them individually as mosaic or germline.

### Mosaic mutations found in blood-derived DNA with MosaicHunter

We also employed MosaicHunter, which uses a Bayesian genotyping algorithm with a series of stringent filters (see Supplemental Methods) for discovering mosaic variants using WGS genotype information from trios [37]. Among the 2530 CHD trios, MosaicHunter identified an initial set of 58,976 sites showing evidence of mosaicism, including 214 high-confidence variants located in coding regions (Fig. 1). After applying a minimum likelihood ratio (LR) cutoff of 80 for distinguishing mosaic from germline mutation, and additional heuristic filters (Supplemental Methods), MosaicHunter identified 116 coding sites (Additional file 1: Table S4) or 0.05 mosaics /individual.

Candidate mosaic variants were compared between the EM-mosaic and MosaicHunter pipelines. Of the mosaic candidates detected by MosaicHunter, 58/116 (50%) were also identified by EM-mosaic while 58/116 (50%) candidates were unique to MosaicHunter (Table 1; Additional file 2: Figure S2). Of the 58 candidates unique to MosaicHunter, 35 were filtered out by EM-mosaic on the basis of insufficient alternate allele read support, 16 had a non-zero allelic depth in the parents, and 7 failed quality filters. The 251 candidates unique to EM-mosaic were discarded by the MosaicHunter pipeline during BAM reprocessing (*n* = 13), quality filtering (*n* = 146), and application of LR cutoff (22), or were not called due to inadequate read depth (*n* = 70) (Fig. 1).

### Sequence confirmation of candidate mosaic variants

Candidate mosaic variants from the EM-mosaic and MosaicHunter were combined into a single list for further evaluation. From the 367 high-confidence EM-mosaic and/or MosaicHunter mosaic SNVs, we selected 143 candidates (75 uniquely identified by EM-mosaic; 46 uniquely identified by MosaicHunter; 22 identified by both) for experimental confirmation using MiSeq amplicon resequencing (Table 1; Additional file 1: Table S5; Additional file 1: Table S11; Additional file 1: Table S12; “Methods”). DNA fragments encompassing the putative mosaic variant were PCR-amplified from proband and each parent DNA, sequenced on an Illumina MiSeq next-generation sequencer and VAF was calculated for each individual. These candidate mosaics included SNVs on the extremes of the VAF spectrum, as well as mosaics that were flagged by MosaicHunter quality filters. In total, we confirmed 108 of 143 candidates as mosaic (Additional file 2: Figure S3A-B), including 21/22 (95%) sites identified by both pipelines. Candidate variants were considered confirmed by MiSeq analyses if they demonstrated an amplicon VAF exceeding 0.01 but less than 0.45, so as to indicate a variant of post-zygotic origin. MiSeq VAF values closely correlated with those originally determined by exome sequencing (*P* = 2.2 × 10^− 16^). Average MiSeq sequencing read depth was 4639 among all candidates and 4354 among confirmed mosaics.

Based on MiSeq VAF values, we confirmed 85/97 (88%) of EM-mosaic candidate mosaic variants with a mean read depth of 4460 (Additional file 2: Figure S3A,C). Three candidate variants were likely germline de novo SNVs (VAF > 0.45). Nine candidate variants were “false positives” that were neither germline de novo SNVs nor mosaic SNVs since either no variant reads were detected by MiSeq sequencing of the proband amplicon, or the same small fraction of variants were detected in proband amplicon and one parent’s amplicon.

Parallel analyses with MosaicHunter confirmed 44/68 (65%) candidate mosaic variants with a mean read depth of 4505 (Additional file 2: Figure S3B,D). There were 23 sites for which no variant reads were detected by MiSeq amplicon sequencing (MiSeq VAF < 0.001) or in which the same small fraction of variant reads was detected in the proband amplicon as in one parent’s amplicon.

### Mosaic detection power calibrated by sequencing depth and estimated true frequency of mosaicism

To better characterize how sequencing data parameters affect the detection of mosaic variants, we considered whether estimates of mosaic variant frequency were sensitive to whole exome sequencing depth by calibrating estimates of mosaic detection power using properties of the sequence data (average read depth, prior mosaic fraction, and the value of our overdispersion parameter *θ*) (Additional file 2: Figure S4; Supplemental Methods)*.* Our projected mosaic detection power curves demonstrated more than a doubling of power to detect mosaic variants with VAF 0.2 as sequencing depth increases from 40× to 80× (Fig. 2c). Projected mosaic detection power curves for less stringent mosaic cutoffs showed similar increases of power with increasing sequencing depth (Additional file 2: Figure S6).

Next, to estimate the “true” frequency of mosaicism per blood DNA exome, independent of average coverage detection power constraints, we estimated the “true” mosaic count in a VAF range by multiplying the number of mosaics by the inverse of the detection power for each VAF bin. Applying this method to the 184 of 309 high-confidence EM-mosaic variants with VAF > 0.1, we estimated the adjusted number of mosaics with VAF > 0.1 to be 361 (Additional file 2: Figure S6A, below). Thus, the true frequency of coding mosaics in the blood (0.4 > VAF > 0.1) is 0.14 variants per individual, representing a non-negligible class of mutations with potential contribution to genetic risk for congenital heart disease. The estimated true mosaic frequency does not change significantly when using less stringent mosaic definitions (Additional file 2: Figure S6, below). In sum, after excluding sites failing MiSeq confirmation, we identified 332 blood mosaic variants in 2530 CHD probands (Table 1) or 0.13 mosaic variants per subject with a mean VAF of 0.13 ± 0.06. We do not anticipate that doubling the sequencing depth would significantly change this estimate—our estimated true frequency of mosaicism above 10% allele fraction (assuming full detection power) in the coding region was 0.14 per subject.

### Mosaic variants occurred most frequently at CpG sequences

**Fig. 3:**
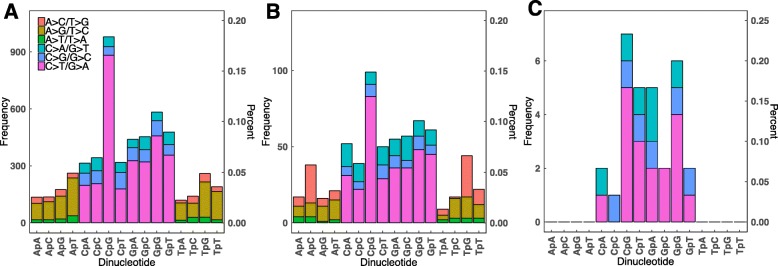
Mutation spectrum of detected germline and mosaic variants. Rates of specific mutations were compared in **a** germline, **b** blood mosaic, and **c** cardiac tissue mosaic variants. Transitions predominated in both variant sets

The nucleotides surrounding candidate mosaic variant mutations were examined to identify whether any dinucleotide sequences were more likely to be associated with mosaicism. Previous studies demonstrated a strong preference for de novo C>T mutations at CpG dinucleotides compared to other dinucleotides due to the spontaneous deamination of 5-methylcytosine [22, 24]. We asked whether the germline de novo variants observed in CHD probands and the 332 mosaic sites demonstrated a similar sequence preference (Fig. 3; Table 1; Additional file 1: Table S3; Additional file 1: Table S4). Of the 2662 germline de novo mutations identified in 2530 CHD probands, 979 variants (37% of all variants) involved mutation of the cytosine of a CpG dinucleotide (Fig. 3a). By contrast, 99 (29% of all mosaic SNVs) of 332 mosaic SNVs altered the cytosine of a CpG dinucleotide; significantly more than expected by chance (2.2× above expectation; *P* = 2 × 10^− 15^). These observations suggest that somatic de novo mutations were 1.4-fold less likely to involve a CpG dinucleotide than germline de novo mutations in CHD probands (*P* = 0.01; Fig. 3b). Even ignoring the high CpG mutation frequency, cytosines and guanines were ~ 2-fold more likely to be mutated than adenines or thymidines both for germline mutations and for mosaic variants. Surprisingly, somatic mutations of A>C/T>G transversions in ApC dinucleotides were ~ 2-fold greater than the corresponding germline mutations (*P* = 5 × 10^− 8^; Fig. 3b). Dinucleotide frequencies for mosaic variants detected in cardiac tissue DNA are shown in Fig. 3c.

### Detection of mosaic mutations in CHD tissues

In addition to exome data from blood or saliva samples, a subset of participants also had exome sequencing data available from cardiac tissue. Using EM-mosaic and MosaicHunter, we analyzed exome sequences from 70 cardiac tissues derived from 66 subjects with CHD (Additional file 1: Table S6) and paired blood samples. Among 57 de novo variants (allele fraction approximately 0.5) that were previously identified in blood-derived DNA, 54 were also found in CHD tissues. Of the 3 de novo variants not present in cardiac tissue, 1 was outside of the tissue WES capture region and 2 occurred in a single proband (Table 2). In addition, 23 distinct candidate mosaic variants were detected by EM-mosaic (*n* = 13), MosaicHunter (*n* = 6), or by both algorithms (*n* = 4). We also detected 5 mosaic candidates in blood WES data that had non-zero read depth in the cardiac tissue WES data corresponding to the same individual but below our minimum alternate allele read depth requirement. All 28 candidates identified in either blood or cardiac tissue were tested via MiSeq amplicon sequencing using both blood and cardiac tissue DNA; 15 of 28 (57%) unique candidate mosaics were confirmed (Table 2; Additional file 1: Table S7), including a *CCNC* variant that was identified in two different cardiac tissues from proband 1-01684 and an *RRS1* variant identified in two different cardiac tissues in proband 1-07299. Ten of 15 (66%) confirmed mosaic variants were detected in blood and cardiac tissues (VAF > 0.01), four were found only in cardiac tissue, and one was found only in blood. Of the 7 mosaics detected by blood WES analysis, 4 were confirmed in the corresponding cardiac tissue sample. Remarkably, five confirmed cardiac tissue mosaic variants occurred in one proband (1-07004), one of which was also present in blood DNA.

**Table 2:** Mosaics detected in individuals with matched cardiovascular tissue and blood

**ID**	**Gene**	**Variant class**	**Pipeline**	**CHD tissue**				**Blood WES VAF**		
				**Location**	**WES AD**	**WES VAF**	**MiSeq VAF**	**WES AD**	**WES VAF**	**MiSeq VAF**
1-00543	*CTCFL*	Bmis	EM-mosaic	AO	138,36	0.21	0.32	29,8	0.22	0.19
1-00984	*ZNF16*	syn	EM-mosaic	LV	262,1	0.00	0.01	100,7	0.07	0.07
1-01282	*GABRA6*	Dmis	MosaicHunter	RV	104,1	0.01	0.01	55,12	0.18	0.18
1-01684	*CCNC*	Bmis	Both	AoValve, RV	36,7	0.16	0.17, 0.19	224,40	0.15	0.14
1-02672	*TOR1A*	syn	Both	AtrSpt	159,10	0.06	0.10	29,6	0.17	0.19
1-03512	*RFX3*	LoF	MosaicHunter	RV	156,15	0.09	0.08	39,0	0.00	0.03
1-04652	*PCDH10*	syn	Both	AtrSpt	154,19	0.11	0.14	15,1	0.06	0.10
1-07004	*ANK2*	Bmis	MosaicHunter	SubAoMembr	226,13	0.05	0.04	30,0	0.00	0.00
1-07004	*MYH14*	Bmis	Both	SubAoMembr	124,22	0.15	0.27	33,0	0.00	0.00
1-07004	*NRG3*	Bmis	EM-mosaic	SubAoMembr	152,30	0.16	0.24	43,0	0.00	0.00
1-07004	*NUDT21*	Bmis	Both	SubAoMembr	137,22	0.14	0.14	74,0	0.00	0.02
1-07004	*TET3*	Dmis	MosaicHunter	SubAoMembr	131,1	0.01	0.03	81,16	0.16	0.27
1-07299	*RRS1*	syn	Both	RV, UNK	160,25	0.14	0.25	22,2	0.08	0.14
1-09869	*PIK3C2G*	LoF	MosaicHunter	LV	126,9	0.07	0.10	31,0	0.00	0.00
1-11800	*TMEM45A*	Bmis	MosaicHunter	RV	213,0	0.00	0.00	32,7	0.18	0.06

Characteristics of mosaic variants predicted for individuals with blood and cardiovascular tissue WES data available. Among 15 mosaics, 5 were detected via analysis of blood WES, 8 were detected from cardiovascular tissue WES, and 2 were detected by both approaches. Six of 7 (86%) mosaics detected from analysis of blood were present in both DNA sources with MiSeq VAF ≥ 0.01. Two additional variants previously identified as de novo germline variants in blood WES were absent from CHD tissue WES. Minimum 1023 MiSeq reads used to determine VAF. Note: multiple cardiovascular tissue samples were available for participants 1-01684 and 1-07299. Abbreviations: *AD* allelic depth (reference, alternate), *AO* aorta, *AtrSpt* atrial septum, *Bmis* benign missense, *Dmis* deleterious missense, *LOF* loss of function variant, *LV* left ventricle, *RV* right ventricle, *VAF* variant allele fraction

**Fig. 4:**
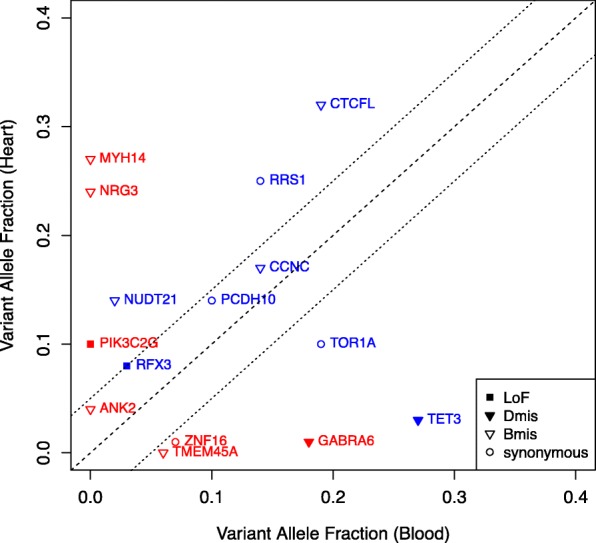
Validated mosaics detected in probands with matched blood and cardiovascular tissue samples available. Validation VAF from blood compared to validation VAF from cardiovascular tissue demonstrated tissue-specific mosaicism (red) as well as shared mosaicism (blue). Predicted effect of mosaic variants corresponds to marker shape

These analyses indicate an observed frequency of coding mosaics in the cardiac tissues of 0.23 per individual (15 confirmed mosaics among 66 probands). In order to estimate the true frequency of mosaicism, we applied the same power-based adjustment approach to the 12 of 15 mosaics with allele fraction greater than 10%. Considering the increased sequencing depth (150×) in our cardiac tissue samples, our estimated true frequency of coding mosaics with VAF > 0.1 is 0.21 per individual (14 mosaics among 66 probands; 2 confirmed mosaics with VAF > 0.1 + 2 additional mosaics assuming full detection power). Finally, comparing the allele fraction of mosaics detected in probands with both blood and cardiac tissue available, we found that mosaics with higher VAF in blood were more likely to be found in both tissues (Mann-Whitney *U* test *P* = 0.019), presumably indicating that the mutation occurred earlier in lineage development (Fig. 4; Additional file 1: Table S7).

### Blood and cardiac tissue mosaics likely to contribute to CHD

Our prior genetic studies of CHD studies showed that damaging de novo variants typically occurred in genes highly expressed in the top quartile of the developing E9.5 mouse heart (HHE) [34, 86] or contributed to CHD in mouse models [42]. Among the 347 mosaic variants identified from blood or cardiac tissue analyses that were not false by MiSeq, 65 altered these HHE and/or mouse CHD genes (*n* = 4558; Additional file 1: Table S8). RefSeq functional annotation predicted 52 variants as likely damaging variants (LOF, Dmis), and 46 as likely benign, missense (Additional file 1: Table S8; Additional file 1: Table S9). In total, we observed potentially CHD-causing mosaic mutations in 25 participants, representing 1% of the 2530 total participants in our CHD cohort. Among these 25 mosaics, we confirmed 22/22 (100%) candidates tested via MiSeq. Notably, multiple likely damaging mosaic variants altered genes (*ISL1*, *SETD2*, *NOVA2*, *SMAD9*, *LZTR1*, *KCTD10*, *KCTD20*, *FZD5,* and *QKI*) involved in key developmental pathways, which may account for the extracardiac phenotypes observed in these patients (Table 3; Additional file 1: Table S10). There was no difference in the proportion of individuals with extracardiac features among those with damaging mosaic variants compared to the overall cohort (11/25 vs 909/2521, *P* = 0.68), and there was a wide range of CHD subtypes. Among genes harboring multiple mosaic variants, none carried more than one mosaic mutation predicted to be damaging (Additional file 1: Table S13). Eight genes were found to harbor one damaging (LoF or Dmis) mosaic mutations and at least one damaging germline variant (Additional file 1: Table S14). Three of the eight genes have more than one damaging germline variants. Among these, *FBN1* and *LZTR1* are well-known risk genes implicated with syndromes that include heart defects. *WASHC5* has been implicated with Ritscher-Schinzel syndrome under a recessive model in an isolated community [18], with CHD as one of the main clinical features. In our cohort, having two damaging germline and one mosaic mutations supports *WASHC5* to be a candidate CHD gene. No CNVs were detected in these subjects, with the exception of 1-00192 (duplication at chr15:22062306-23062355; non-overlapping with the *GLYR1* mosaic).

**Table 3:** Damaging mosaics in CHD-relevant genes

**ID**	**Gene**	**Variant class**	**Blood VAF**	**pLI**	**Episcore**	**HeartExp**	**Age (year)**	**Clinical phenotype**		**PCGC de novo LoF/Dmis variants in mosaic gene**
								**Cardiac abnormalities**	**Extracardiac abnormalities**	
1-00761	*FBN1*	Dmis	0.24	1.00	98	93	1–5	Mitral stenosis	Dysmorphic features, subglottic stenosis, hypoplasic left mainstem bronchus, short stature	3
1-07004	*TET3*	Dmis	0.16	1.00	7	87	5–18	Subaortic stenosis	None	0
1-05662	*SETD2*	LoF	0.13	1.00	99	85	< 1	Aortic coarctation, mitral valve hypoplasia	None	0
1-00344	*UBR5*	splice	0.27	1.00	95	90	5–18	D-transposition of the great arteries, VSD, valvar and subvalvar pulmonary stenosis	None	0
1-03512	*RFX3*	LoF	0.09	1.00	100	46	< 1	Tetralogy of Fallot with pulmonary stenosis	None	0
1-06216	*ITSN1*	Dmis	0.21	1.00	98	86	< 1	ASD	Plagiocephaly, rib anomaly, single kidney, dysmorphic facial features	0
1-00363	*QSER1*	Dmis	0.06	1.00	94	79	1–5	Tetralogy of Fallot with pulmonary stenosis, VSD	inguinal hernia	0
1-13185	*PKD1*	Dmis	0.10	1.00	87	84	< 1	VSD, partially anomalous pulmonary venous return	Hemangioma	1
1-00192	*GLYR1*	Dmis	0.22	0.99	89	93	< 1	ASD, VSD, interrupted aortic arch, hypoplastic tricuspid valve, BAV	None	0
1-04046	*FZD5*	Dmis	0.09	0.99	89	48	< 1	Tetralogy of Fallot with pulmonary stenosis, VSD	None	0
1-06649	*NOVA2*	Dmis	0.15	0.95	75	56	< 1	Tetralogy of Fallot with pulmonary stenosis	None	0
1-05095	*ISL1*	LoF	0.07	0.90	97	25	1–5	ASD	None	0
1-06677	*KCTD10*	Dmis	0.16	0.84	75	91	5–18	Aortic coarctation, pulmonary valve stenosis	Dysmorphic facial features, hydrocephalus, pyloric stenosis, single kidney, imperforate/atretic anus	0
1-05447	*HNRNPAB*	Dmis	0.09	0.76	72	99	5–18	ASD, BAV, aortic coarctation	None	0
1-00021	*QKI*	LoF	0.13	0.76	94	97	< 1	Doublet outlet right ventricle, pulmonary stenosis, VSD	None	0
1-11871	*FHOD3*	Dmis	0.18	0.05	91	92	< 1	Tetralogy of Fallot with pulmonary atresia	Hypocalcemia, thrombocytopenia, lymphopenia	0
1-01458	*HK2*	Dmis	0.27	0.04	89	90	< 1	Hypoplastic left heart with aortic and mitral atresia, aortic coarctation	None	1
1-00669	*PRKD3*	splice	0.19	0.02	77	82	< 1	D-transposition of the great arteries, conal VSD, bilateral conus, interrupted aortic arch	None	0
1-00524	*RNF20*	LoF	0.10	0.00	55	83	18–25	Left-dominant complete atrioventricular canal	Heterotaxy with situs inversus totalis, asplenia, duodenal atresia	0
1-01851	*SUCLA2*	LoF	0.11	0.00	72	89	5–18	Balanced complete atrioventricular canal, aortic coarctation	None	0
1-03885	*LZTR1*	Dmis	0.20	0.00	31	84	5–18	Abnormal pulmonary vein draining into the right atrium	Left-sided/midline liver, asplenia, maltrotation	2
1-05011	*KCTD20*	Dmis	0.26	0.00	76	77	18–25	Transposition of the great arteries, tricuspid and pulmonary valve atresia	Left-sided/midline liver	1
1-00018	Figure 4	Dmis	0.19	0.00	49	70	5–18	BAV, mitral atresia, aortic coarctation, VSD, total anomalous pulmonary venous return	Nephritis	1
1-05661	*SMAD9*	Dmis	0.06	0.00	84	39	5–18	Common atrioventricular canal	None	0
1-09869	*PIK3C2G*	LoF	0.07*	0.00	73	28	5–18	Common atrioventricular canal, aortic stenosis, aortic arch hypoplasia, VSDs	Dysmorphic facial features, low-set ears, campomelic dysplasia	1

There were 25 potentially pathogenic mosaic mutations based on known gene function and patient phenotype. Some of these probands have previously described rare LoF/Dmis variants, though none are likely pathogenic for CHD {Jin 2017}. Additionally, some genes were previously found to have LoF/Dmis variants among other individuals in this CHD cohort. Age ranges “A-B” denote A < =age < B. Abbreviations: *ASD* atrial septal defect, *BAV* bicuspid aortic valve, *Dmis* deleterious missense, *episcore* haploinsufficiency score (percentile rank) [29], *Heart Exp* heart expression percentile rank, *LoF* loss of function, *pLI* probability of loss-of-function intolerance, *PCGC* Pediatric Cardiac Genomics Consortium, *VAF* variant allele fraction, *VSD* ventricular septal defect. *VAF refers to CHD tissue WES

**Fig. 5:**
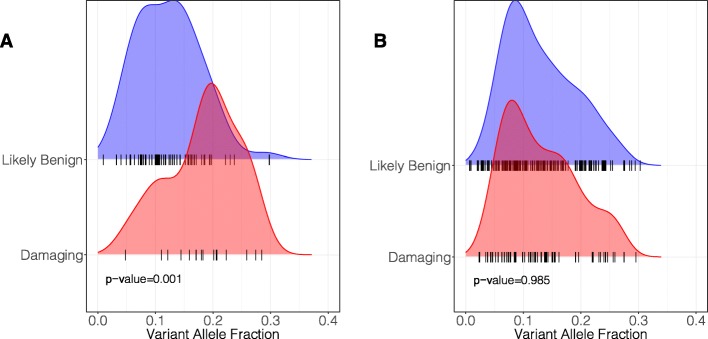
Damaging mosaics in CHD-related genes have higher variant allele fraction than likely benign mosaics. **a** Among the 76 mosaics in CHD-related genes, likely damaging variants have a higher VAF than likely benign (Mann-Whitney *U p* = 0.001). **b** Among the 233 mosaics in other (non-CHD-related) genes, there is no difference in VAF based on predicted effect (*p* = 0.985)

If mosaic variants were unrelated to CHD, we would expect similar allelic fractions between mosaics with variants predicted as likely damaging or likely benign. However, we found that the allele fraction of likely damaging variants in CHD-related genes (union of HHE and mouse CHD genes) was significantly higher (Mann-Whitney *U* test *P* = 0.001; Fig. 5a). Moreover, among mosaic variants in non-CHD-related genes, we found no significant difference in allele fraction (*P* = 0.985; Fig. 5b). We repeated these analyses using (i) less stringent posterior odds cutoffs of 2 and 5 (Figure S8) and (ii) after excluding the 9 mosaics detected from saliva DNA (Additional file 2: Figure S14) and found the same result. Together, these data support our conclusion that at least some likely damaging mosaic variants identified here contribute to CHD. These results were determined independently of MiSeq validation results.

## Discussion

Distinguishing mosaic mutations from constitutional mutations has both clinical management and reproductive implications for proband and parents. Individuals with mosaic mutations are generally clinically less severely affected for conditions that affect multiple parts of the body [8, 15, 19, 30, 82, 84]. Mutations that occur post-zygotically should have no recurrence risk for the parents and could have a recurrence risk of less than 50% for the proband depending on gonadal involvement. This study is among the first investigations of the role of post-zygotic mosaic mutations in CHD. We developed a new computational method to robustly detect mosaic single-nucleotide variants from blood WES data at standard read depth. Contrary to existing methods, EM-mosaic estimates prior mosaic fraction directly from the data instead of using a fixed parameter, which improves our ability to distinguish high allele fraction mosaic mutations from germline mutations. Additionally, our method also uses a stringent filtering approach to remove false positive calls, minimizing their impact on downstream mosaic detection and improving model specificity. Applying this method to a cohort of 2530 CHD patients, EM-mosaic detected 309 high-confidence mosaics (with a confirmation frequency of 88% in a subset of variants assessed) or 0.12 variants per proband. Sequencing of cardiac tissue to greater depth identified an additional 8 mosaic variants that had not been detected in blood WES, 6 of which are present in cardiac tissue but not blood. We found more variants per proband in cardiac tissue DNA (0.23 variants per proband) than in blood DNA (0.13 variants per proband). While the increased numbers of mosaic variants in cardiac tissue DNA vs blood DNA may reflect technical (capture method) differences such as sequencing read depth or coverage uniformity of cardiac tissue DNA vs blood DNA, it is possible that somatic variation occurs more frequently in cardiac tissue of CHD probands than in their blood. Ten of 15 mosaic variants among those identified in our 66 CHD proband cardiac tissues had higher VAF in cardiac tissue than in blood (Table 2) and 5 of 15 variants among these individuals had a higher VAF in blood than in tissue.

In total, we observed potentially CHD-causing mosaic mutations in 25 participants, representing 1% of the 2530 total participants in our CHD cohort. Among these 25 mosaics, we confirmed 22/22 (100%) candidates tested. We found that in CHD-related genes, likely damaging mosaic mutations have significantly greater alternative allele fraction than likely benign mosaics, suggesting that some of these variants contribute to CHD. Comparison of blood and cardiovascular tissues demonstrated tissue-specific mosaic variants, though those variants with a higher VAF were more likely to be shared between tissues. Due to limitations of conventional clinical interpretation for both mosaic and constitutional CHD variants (Supplemental Methods), we cannot know with complete certainty which among these 25 variants is pathogenic and instead propose that, among our detected mosaics, the 23 detected from blood WES data provide an estimate of the disease-causing mosaics detectable in blood with standard exome sequencing read depth. Nine of these variants affect genes known to have a role in cardiac development: *ISL1*, *SETD2*, *NOVA2*, *QKI*, *SMAD9*, *LZTR1*, *KCTD10*, *KCTD20*, and *FZD5*.

The mosaic LoF mutation in *ISL1* is likely to be the cause of CHD in participant 1-05095. *ISL1* is a transcription factor essential to normal cardiac development that regulates expression of *NKX*, *GATA*, and *TBX* family genes [9, 28] and controls secondary heart field differentiation and atrial septation [5, 9]. *ISL1* deficiency has been shown to lead to severe CHD in mice [6, 28]. Participant 1-05095 has an isolated atrial septal defect consistent with a secondary heart field defect phenotype [75] and has no other previously reported damaging germline variants in CHD-related genes.

Damaging germline de novo variants in CHD subjects are enriched in genes related to chromatin modification and RNA processing [34, 42]. Three genes with damaging mosaic variants discovered here have related functions. *SETD2* is a histone methyltransferase required for embryonic vascular remodeling [36]; it is both sensitive to haploinsufficiency and highly expressed in the heart during development. *NOVA2* is a key alternative-splicing regulator involved in angiogenesis that has been shown to disrupt vascular lumen formation when depleted [27]. *QKI* encodes an RNA-binding protein that regulates splicing, RNA export from the nucleus, protein translation, and RNA stability [48]. *QKI* is also highly expressed in the heart during development and has been shown to cause CHD and other blood vessel defects in mice when dysregulated [62].

Other damaging mosaic variants affect processes known to be relevant to CHD. *SMAD9* is involved in the TGF-beta signaling pathway. TGF-beta signaling plays a critical role in cardiac development and cardiovascular physiology, leading to pulmonary arterial hypertension and cardiac abnormalities in mice when dysregulated [17, 74]. *LZTR1* encodes a member of the BTB-Kelch superfamily that is highly expressed in the heart during development and has been associated with Noonan [26, 85] and DiGeorge Syndromes [46], both of which are characterized by CHD. The individual with the *LZTR1* damaging mosaic variant did have pulmonary lymphangiectasias that are a less common feature of Noonan syndrome, but did not have other clinical findings common to Noonan syndrome. *KCTD10* binds to and represses the transcriptional activity of *TBX5* (T-box transcription factor), which plays a dose-dependent role in the formation of cardiac chambers [80]. *KCTD10* is highly expressed in the heart during development and has been shown to produce CHD in mice when dysregulated [67]. *KCTD20* is a positive regulator of *Akt* [61] also highly expressed in the heart during development. *FZD5* is haploinsufficient and encodes a transmembrane receptor involved in Wnt, mTOR, and Hippo signaling pathways and has been shown to play a role in cardiac development [10]. The individual with a damaging mosaic variants in *FBN1*, which is associated with several genetic syndromes, had features consistent with Weill-Marchesani syndrome such as brachycephaly, mitral valve stenosis, short stature, and midface hypoplasia.

Finally, two mosaic variants found in cardiac tissue, genes encoding *RFX3* and *PIK3C2G*, may be disease-relevant. *PIK3C2G* is a signaling kinase involved in cell proliferation, survival, and migration, as well as oncogenic transformation and protein trafficking (OMIM: 609001). The effects of *PIK3C2G* haploinsufficiency during cardiac development have not been characterized. *RFX3* is a highly constrained ciliogenic transcription factor that leads to pronounced laterality defects [65], and disruption of *RFX3* leads to congenital heart malformations in mice (MGI: 5560494) [72]. Notably, the RFX3 LoF variant has a fourfold higher VAF in cardiac tissue than in blood.

Three capture platforms were used in this study. The main technical difference between capture platforms is depth of coverage. We do not believe this to confound the main results of this study. Regarding (1) the estimated rate of mosaic mutations in coding regions—we estimated the rate based on observed number of mosaic mutations normalized by the detection power of such mutations in individual samples. The sequencing depth was a main factor considered in our calculation of detection power. Regarding (2) the genetic contribution of mosaic mutations to CHD—we concluded that mosaic mutations contribute to CHD based on the fact that the damaging mosaic mutations have overall greater allele fraction than benign mosaic mutations in plausible CHD genes. Since capture method and sequencing depth are independent of the type of variants in each gene, we do not expect capture kit to confound this analysis.

Several investigators, who studied cancer and diseases with cutaneous manifestations, proposed that the VAF correlates with time of mutation acquisition and disease burden [3, 31, 71]. In this study, we used VAF as a proxy for cellular percentage and mutational timing, with increasing VAF corresponding to events occurring earlier in development. Thus, we assume that CHD-associated mosaic events identified in blood-derived DNA occurred during or shortly after the gastrulation process (third week of development) [60] in the mesodermal progenitor cells that differentiate into both heart precursor cells (cardiogenic mesoderm) and blood precursor cells (hemangioblasts). We found that in CHD-relevant genes, mosaic sites predicted to be damaging tended to have higher VAF than sites predicted to be likely benign, consistent with the hypothesis that these mutations arose early in fetal development and play significant roles in CHD. However, additional functional studies are necessary to fully assess causality.

We recognize that while our method is able to detect a large fraction of mosaic variants in blood, our calibrated estimates for the true number of mosaics suggest there are a non-negligible number of additional mutations that were not identified by our method. At our current average sequencing depth of 60×, we have limited sensitivity in the low VAF (< 0.05) range. To reliably identify these low allelic fraction sites, ultra-deep sequencing will be critical to distinguishing true variants from noise. At 500×, we estimate detection sensitivity for mosaic events at VAF 0.05 to be above 80%. Additionally, copy number variations (CNVs) are well documented as contributors to CHD [87] and somatic CNVs comprise a class of potentially impactful mosaic events. However, neither of the methods presented are designed to detect mosaic CNVs since the computational problem of detecting mosaic CNVs is very different to detecting mosaic SNV/indels. We also recognize age-related clonal hematopoiesis [25, 40] as a potential confounding factor in somatic mutation detection; however, our study cohort includes mostly pediatric cases and we did not observe mosaic mutations in genes related to clonal expansion (e.g., *ASXL1, DNMT3A, TET2, JAK2*) nor did we observe a relationship between proband age and mosaic rate (Additional file 2: Figure S9), suggesting minimal impact from this process. We also did not find evidence of a relationship between parental age (paternal or maternal) and proband mosaicism (Additional file 1: Table S16; Additional file 2: Figure S10; Additional file 2: Figure S11).

In this manuscript, we presented the results of our case-only analysis due to the lack of appropriate controls. To allow direct comparison, controls would need to be matched on the basis of age, sex, sequencing depth, and DNA source (blood). While at the time of this study such controls were not available, recent efforts to promote data sharing and availability may yield an appropriate set of controls in the near future and enable estimating the contribution of mosaicism to CHD with higher resolution and certainty.

## Conclusions

This study is among the first investigations of the role of post-zygotic mosaic mutations in CHD. Despite limitations in sequencing depth and sample type, EM-mosaic was able to detect 309 high-confidence mosaics from blood, with resequencing confirmation in 88% of cases assessed, and 17 candidates in cardiac tissue (41% confirmation rate). Using MosaicHunter, an additional 64 candidate mosaic sites were identified, of which 23/46 (50%) candidates from blood DNA and 4/6 (67%) from CHD tissue DNA validated. We observed mosaic frequencies of 0.13/individual in blood and 0.23/individual in cardiac tissue. Assuming full detection power, we estimate the true frequency of mosaic variants in the coding region above 10% mosaicism to be 0.14/individual in blood and 0.26/individual in cardiac tissue. In total, we observed potentially CHD-causing mosaic mutations in 25 participants, representing 1% of our CHD cohort, and propose that these 25 cases provide an estimate of the disease-causing mosaics detectable in blood with standard exome sequencing read depth. Additionally, we found that in CHD-related genes, likely damaging mosaics have significantly greater alternative allele fraction than likely benign mosaics, suggesting that many of these variants cause CHD and occurred early in development. In the subset of our cohort for which cardiovascular tissue samples were available, we show that mosaics detected in blood can also be found in the disease-relevant tissue and that, while the VAF for mosaic variants often differed between blood and cardiovascular tissue DNA, variants with higher VAF were more likely to be shared between tissues. Given current limitations in sequencing depth and on the availability of relevant tissues, particularly for conditions impacting internal organs like the heart, the full extent of the role of mosaicism in many diseases remains to be explored. However, as datasets containing larger numbers of blood and other tissue samples sequenced at higher depths become increasingly available, we will be able to more fully characterize the biological processes underlying post-zygotic mutation and, by extension, the contribution of mosaicism to disease using the methods presented here.

## Additional files

Additional file 1. Contains Supplemental Tables S1-S16.

Additional file 2. Contains Supplemental Figures S1-S14.

Additional file 3. Contains Supplemental Methods.

## Data Availability

EM-mosaic and custom scripts and pipeline for analyzing data are available from https://github.com/ShenLab/mosaicism [34]. The MosaicHunter software is available from http://mosaichunter.cbi.pku.edu.cn/ [36]. SAMtools is available from http://www.htslib.org/ [52]. ANNOVAR is available from http://annovar.openbioinformatics.org/en/latest/ [85]. Integrative Genomics Viewer (IGV) software is available from https://software.broadinstitute.org/software/igv/ [70]. Whole exome sequencing data have been deposited in the database of Genotypes and Phenotypes (dbGaP) under accession numbers phs000571.v1.p1 [11], phs000571.v2.p1 [12], and phs000571.v3.p2 [13].

## Abbreviations

ASD: Autism spectrum disorder
CHD: Congenital heart disease
dnSNV: De novo SNV
Dmis: Deleterious missense mutation
DP_site_: Total read depth at a variant site
DP_sample_: Sample-wide average read depth
ExAC: Exome Aggregation Consortium
FDR: False discovery rate
gnomAD: Genome Aggregation Database
HHE: High heart expression
LOF: Loss of function
LR: Likelihood ratio
MAF: Minor allele frequency
*N*: Total read depth
*N*_alt_: Alternate allele read depth
OR: Odds ratio
PCGC: Pediatric Cardiac Genomics Consortium
pLI: Probability of loss-of-function intolerance
PV4: *P* value for strand bias, baseQ bias, mapQ bias and tail distance bias (SAMtools)
SNV: Single-nucleotide variant
VAF: Variant allele frequency
WES: Whole exome sequencing
