# Supplementary material for: EM-mosaic detects mosaic point mutations that contribute to congenital heart disease
Source: Genome Med. 2020 Apr 29;12:42. doi: 10.1186/s13073-020-00738-1 (PMC7189690; doi:10.1186/s13073-020-00738-1)
Supplement: Supplementary file 2 — Contains Supplemental Figures S1-S14. [file 13073_2020_738_MOESM2_ESM.pptx]

## Slide 1
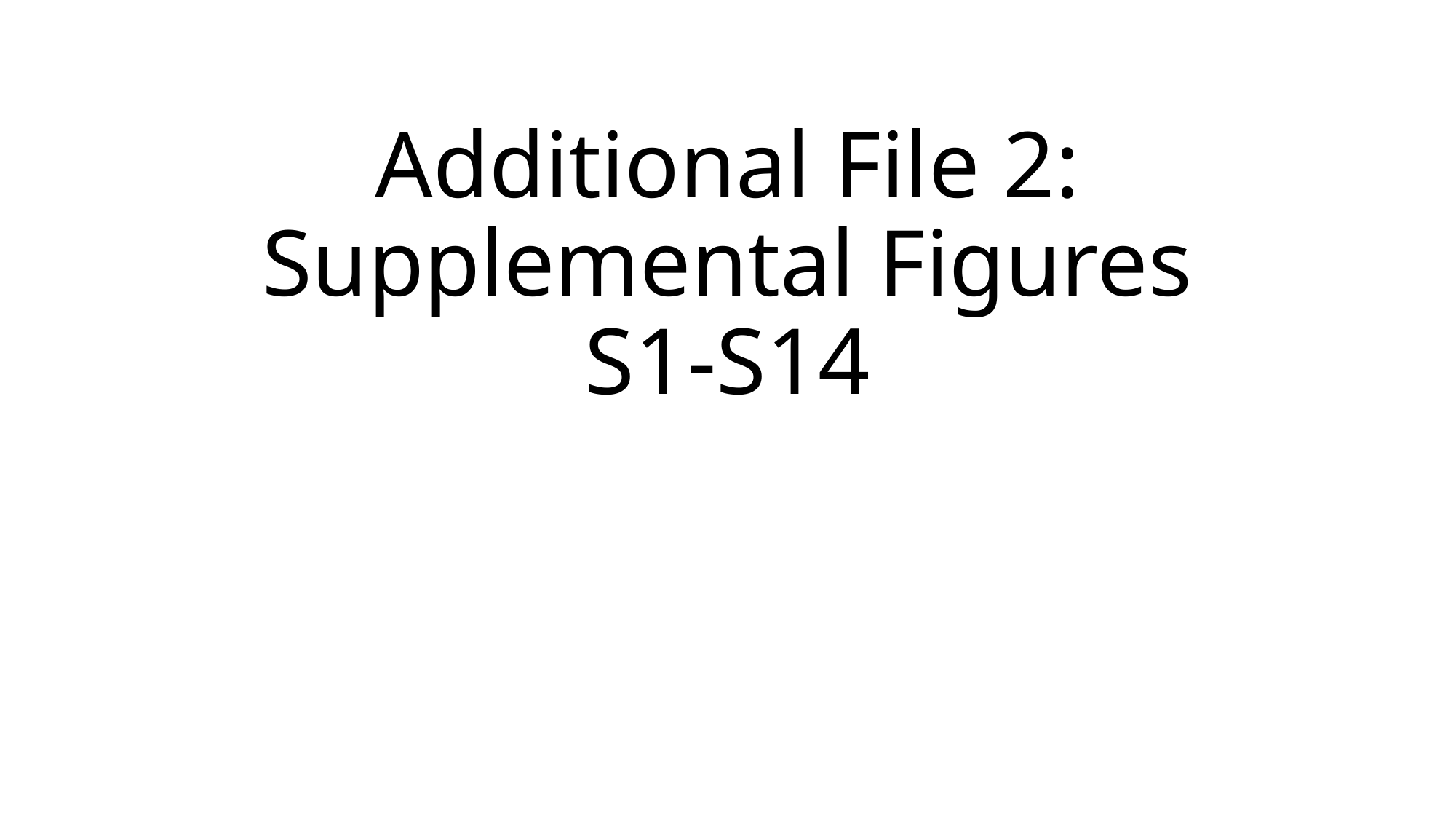

# Additional File 2: Supplemental Figures S1-S14

## Slide 2
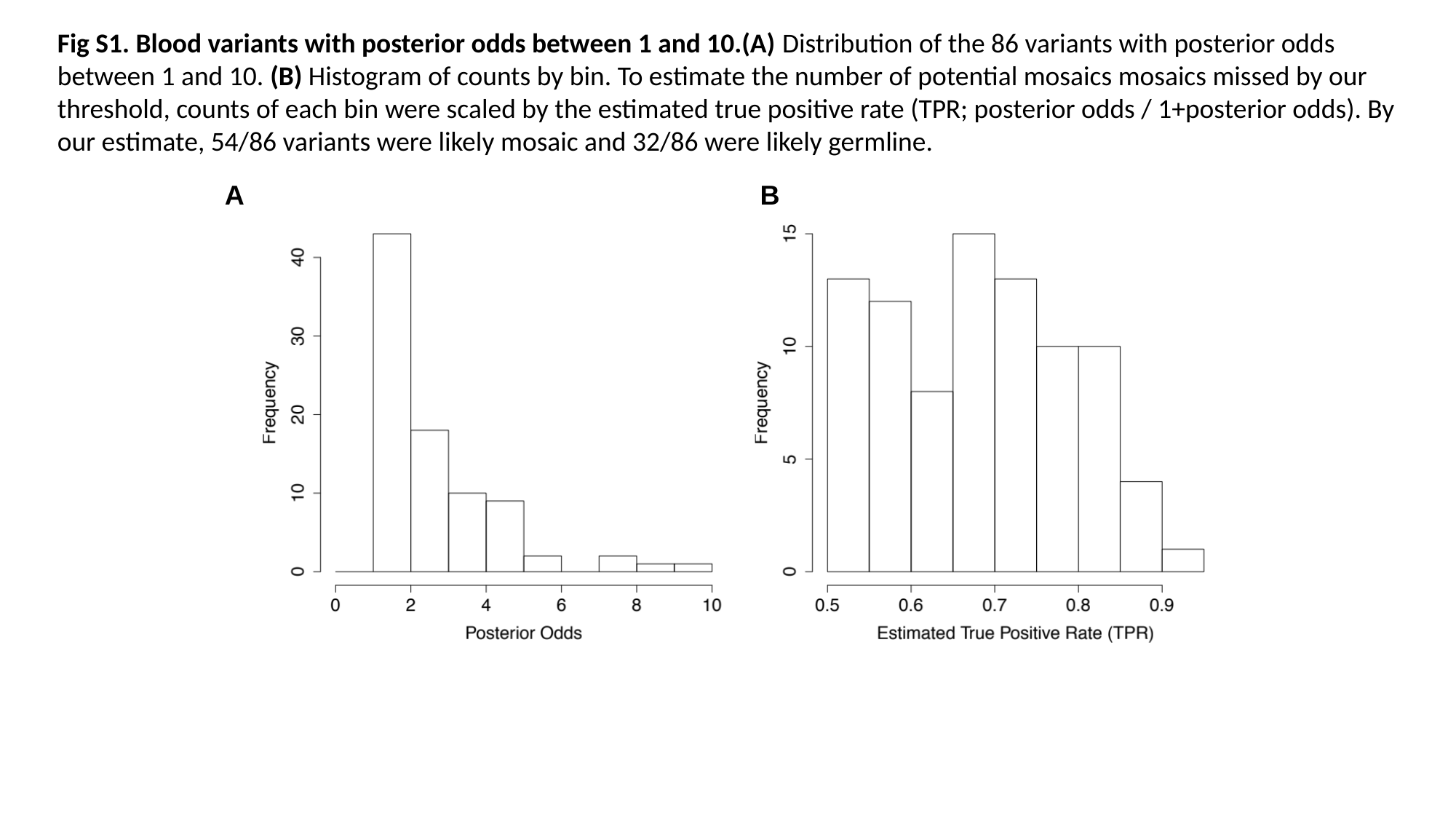

Fig S1. Blood variants with posterior odds between 1 and 10.(A) Distribution of the 86 variants with posterior odds between 1 and 10. (B) Histogram of counts by bin. To estimate the number of potential mosaics mosaics missed by our threshold, counts of each bin were scaled by the estimated true positive rate (TPR; posterior odds / 1+posterior odds). By our estimate, 54/86 variants were likely mosaic and 32/86 were likely germline.
A
B

## Slide 3
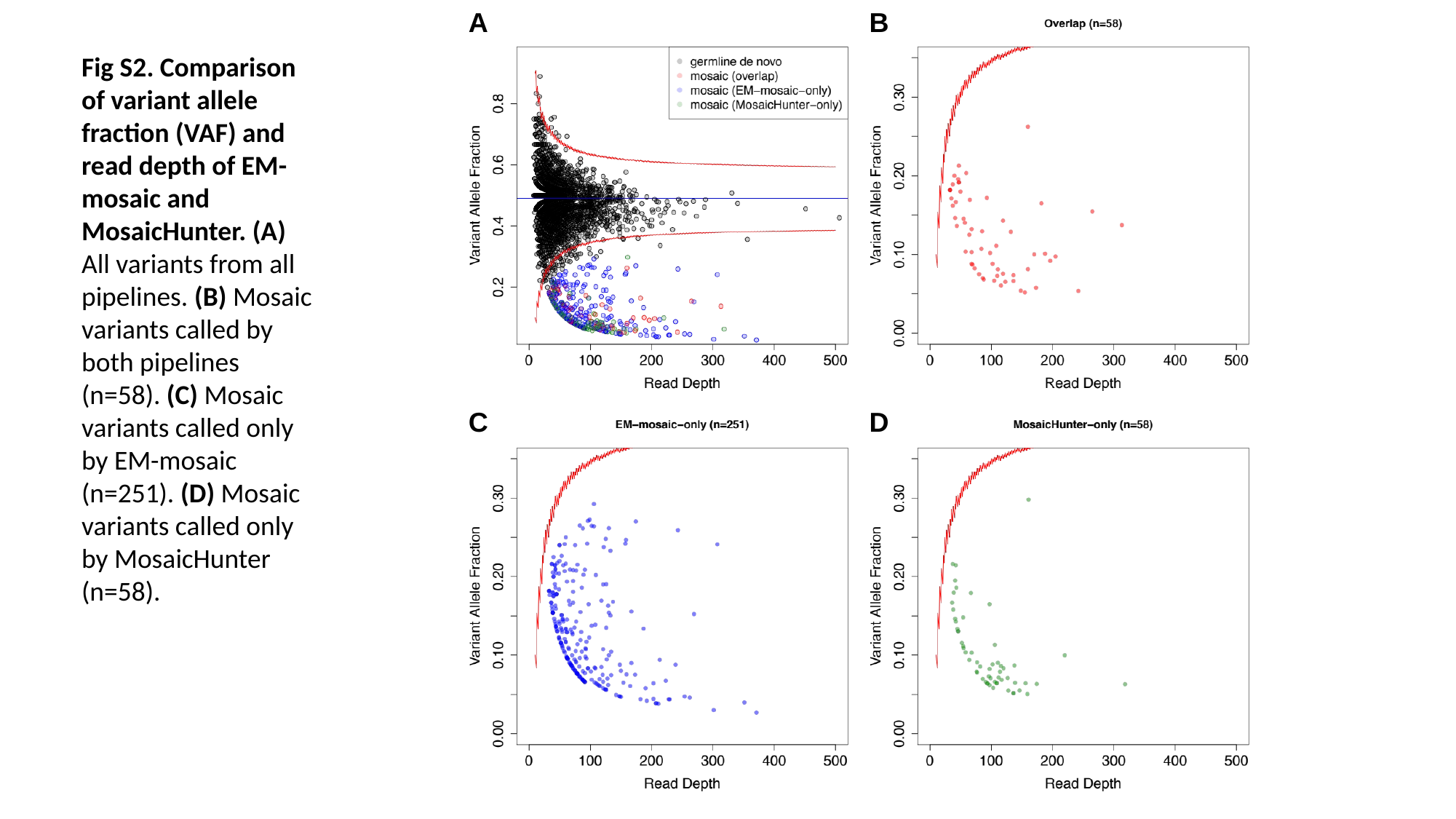

A
B
C
D
Fig S2. Comparison of variant allele fraction (VAF) and read depth of EM-mosaic and MosaicHunter. (A) All variants from all pipelines. (B) Mosaic variants called by both pipelines (n=58). (C) Mosaic variants called only by EM-mosaic (n=251). (D) Mosaic variants called only by MosaicHunter (n=58).

## Slide 4
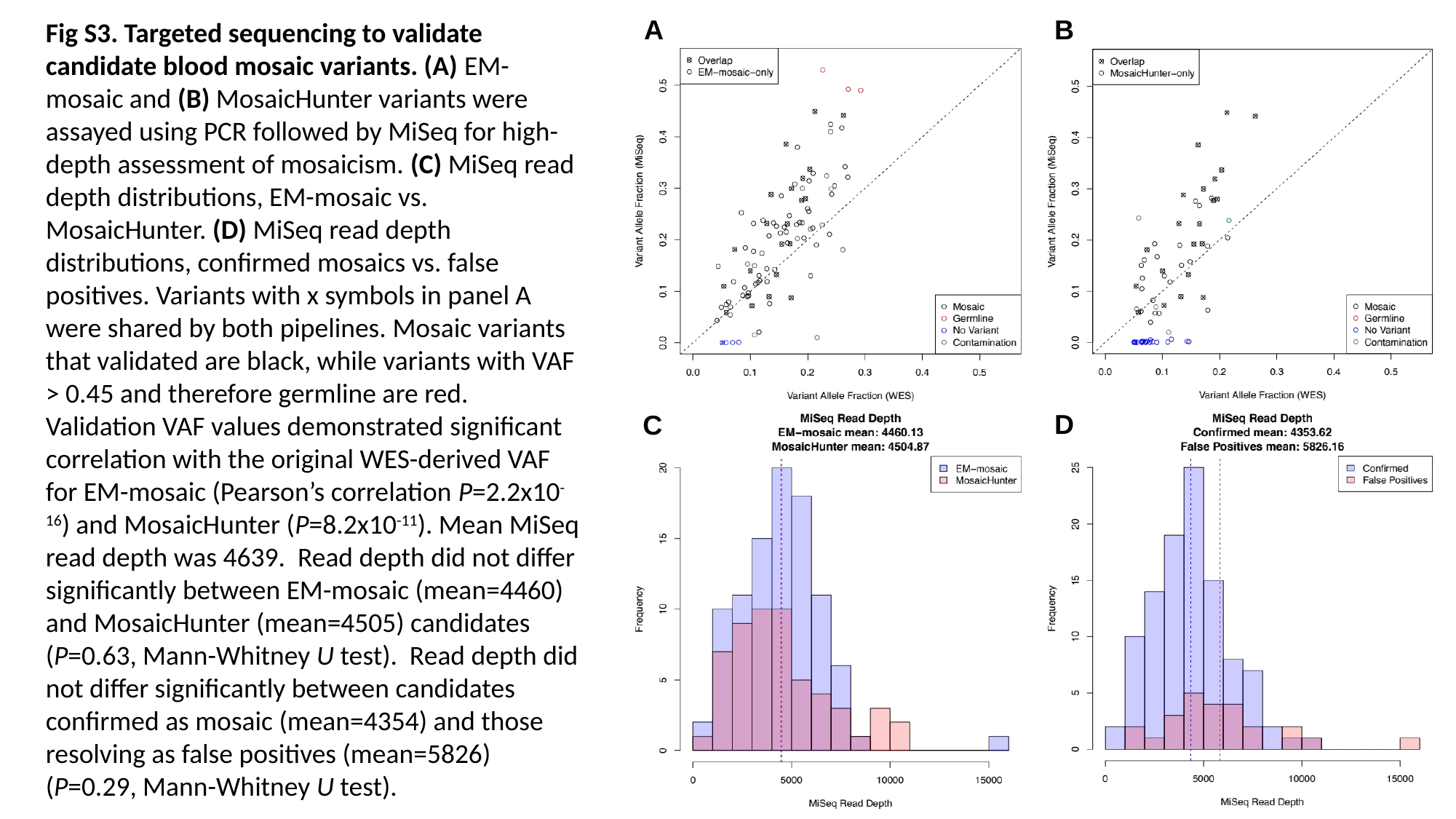

A
B
Fig S3. Targeted sequencing to validate candidate blood mosaic variants. (A) EM-mosaic and (B) MosaicHunter variants were assayed using PCR followed by MiSeq for high-depth assessment of mosaicism. (C) MiSeq read depth distributions, EM-mosaic vs. MosaicHunter. (D) MiSeq read depth distributions, confirmed mosaics vs. false positives. Variants with x symbols in panel A were shared by both pipelines. Mosaic variants that validated are black, while variants with VAF > 0.45 and therefore germline are red. Validation VAF values demonstrated significant correlation with the original WES-derived VAF for EM-mosaic (Pearson’s correlation P=2.2x10^-16^) and MosaicHunter (P=8.2x10^-11^). Mean MiSeq read depth was 4639. Read depth did not differ significantly between EM-mosaic (mean=4460) and MosaicHunter (mean=4505) candidates (P=0.63, Mann-Whitney U test). Read depth did not differ significantly between candidates confirmed as mosaic (mean=4354) and those resolving as false positives (mean=5826) (P=0.29, Mann-Whitney U test).
D
C

## Slide 5
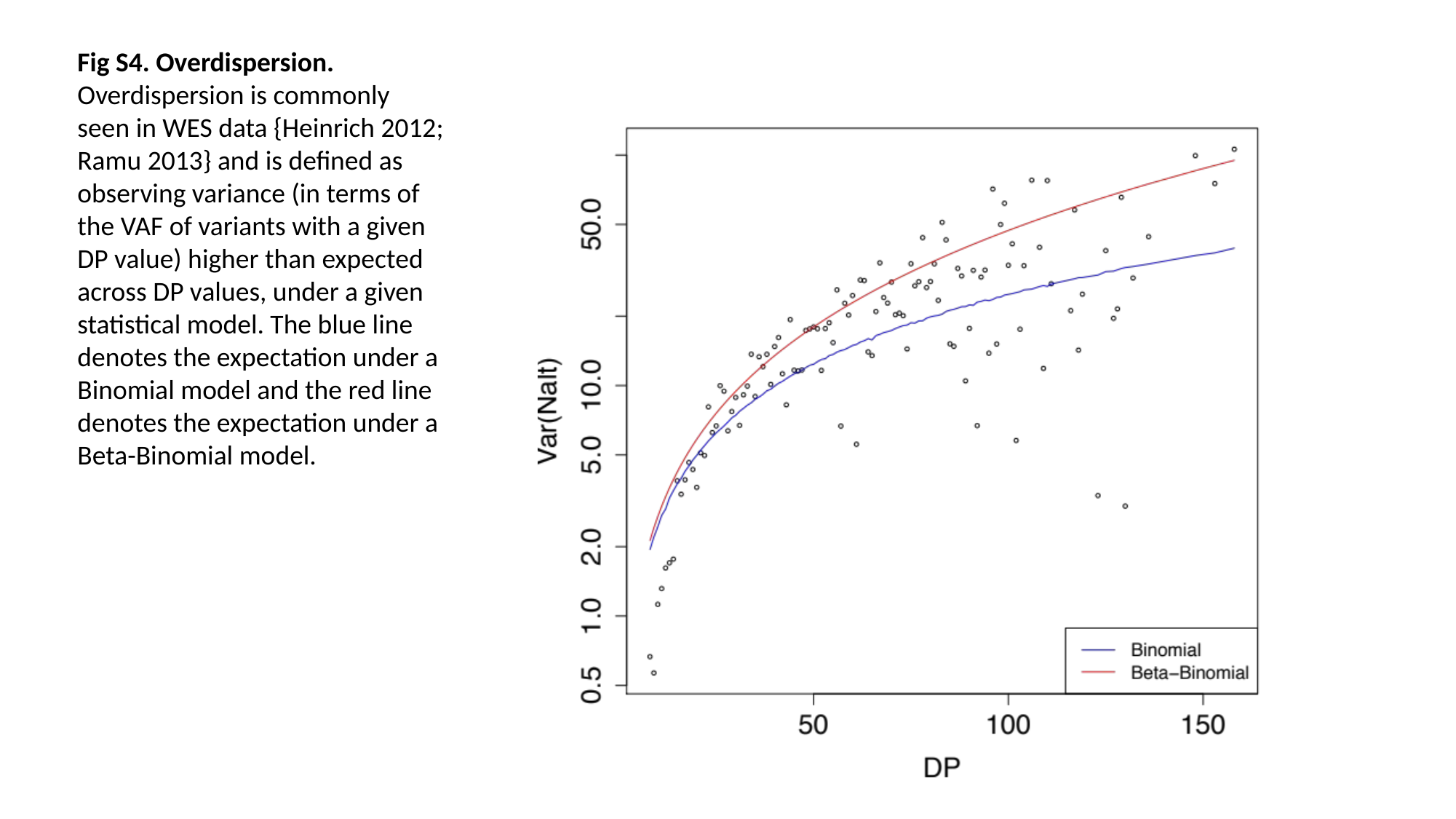

Fig S4. Overdispersion. Overdispersion is commonly seen in WES data {Heinrich 2012; Ramu 2013} and is defined as observing variance (in terms of the VAF of variants with a given DP value) higher than expected across DP values, under a given statistical model. The blue line denotes the expectation under a Binomial model and the red line denotes the expectation under a Beta-Binomial model.

## Slide 6
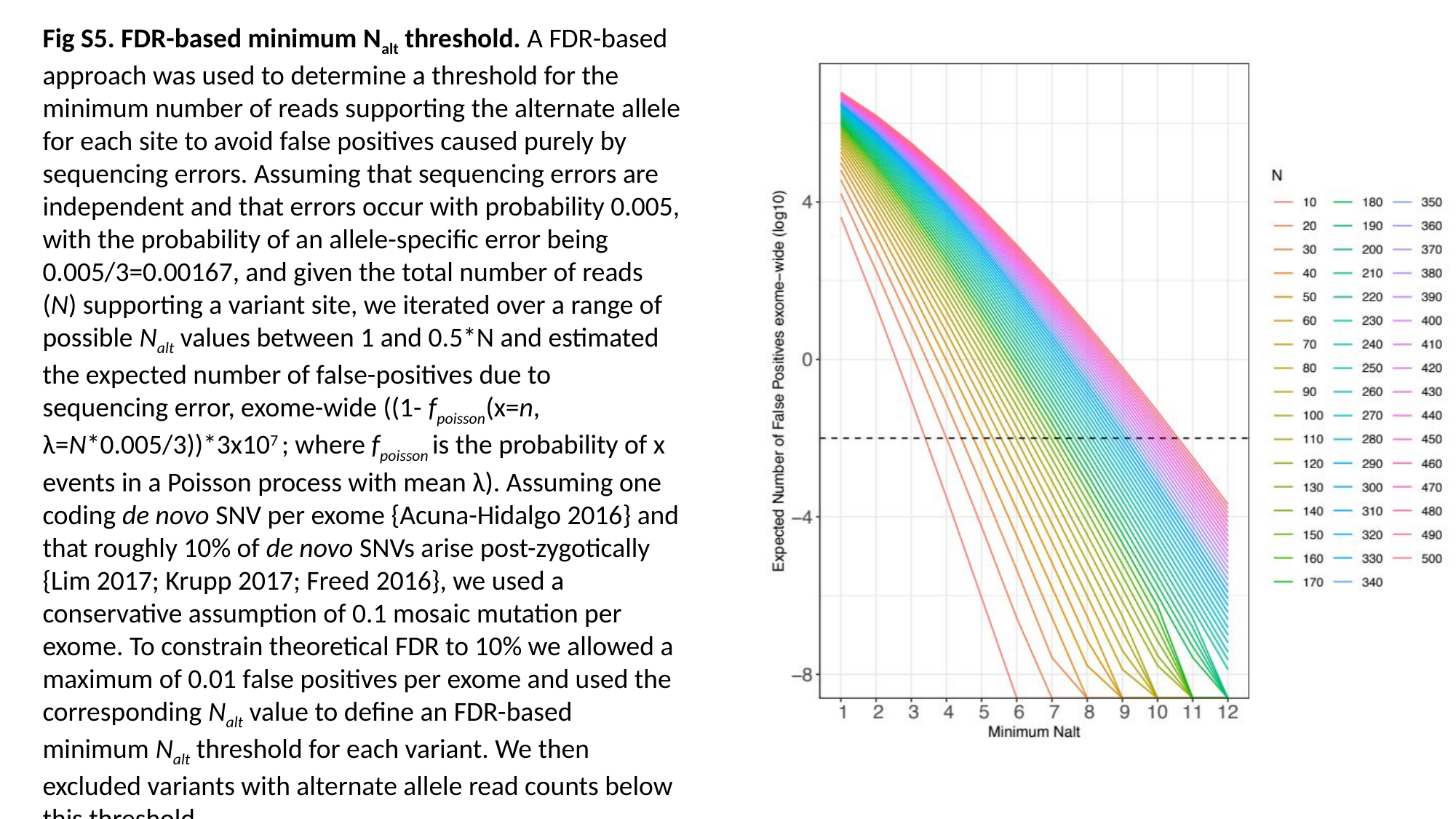

Fig S5. FDR-based minimum Nalt threshold. A FDR-based approach was used to determine a threshold for the minimum number of reads supporting the alternate allele for each site to avoid false positives caused purely by sequencing errors. Assuming that sequencing errors are independent and that errors occur with probability 0.005, with the probability of an allele-specific error being 0.005/3=0.00167, and given the total number of reads (N) supporting a variant site, we iterated over a range of possible Nalt values between 1 and 0.5*N and estimated the expected number of false-positives due to sequencing error, exome-wide ((1- fpoisson(x=n, λ=N*0.005/3))*3x10^7^ ; where fpoisson is the probability of x events in a Poisson process with mean λ). Assuming one coding de novo SNV per exome {Acuna-Hidalgo 2016} and that roughly 10% of de novo SNVs arise post-zygotically {Lim 2017; Krupp 2017; Freed 2016}, we used a conservative assumption of 0.1 mosaic mutation per exome. To constrain theoretical FDR to 10% we allowed a maximum of 0.01 false positives per exome and used the corresponding Nalt value to define an FDR-based minimum Nalt threshold for each variant. We then excluded variants with alternate allele read counts below this threshold.

## Slide 7
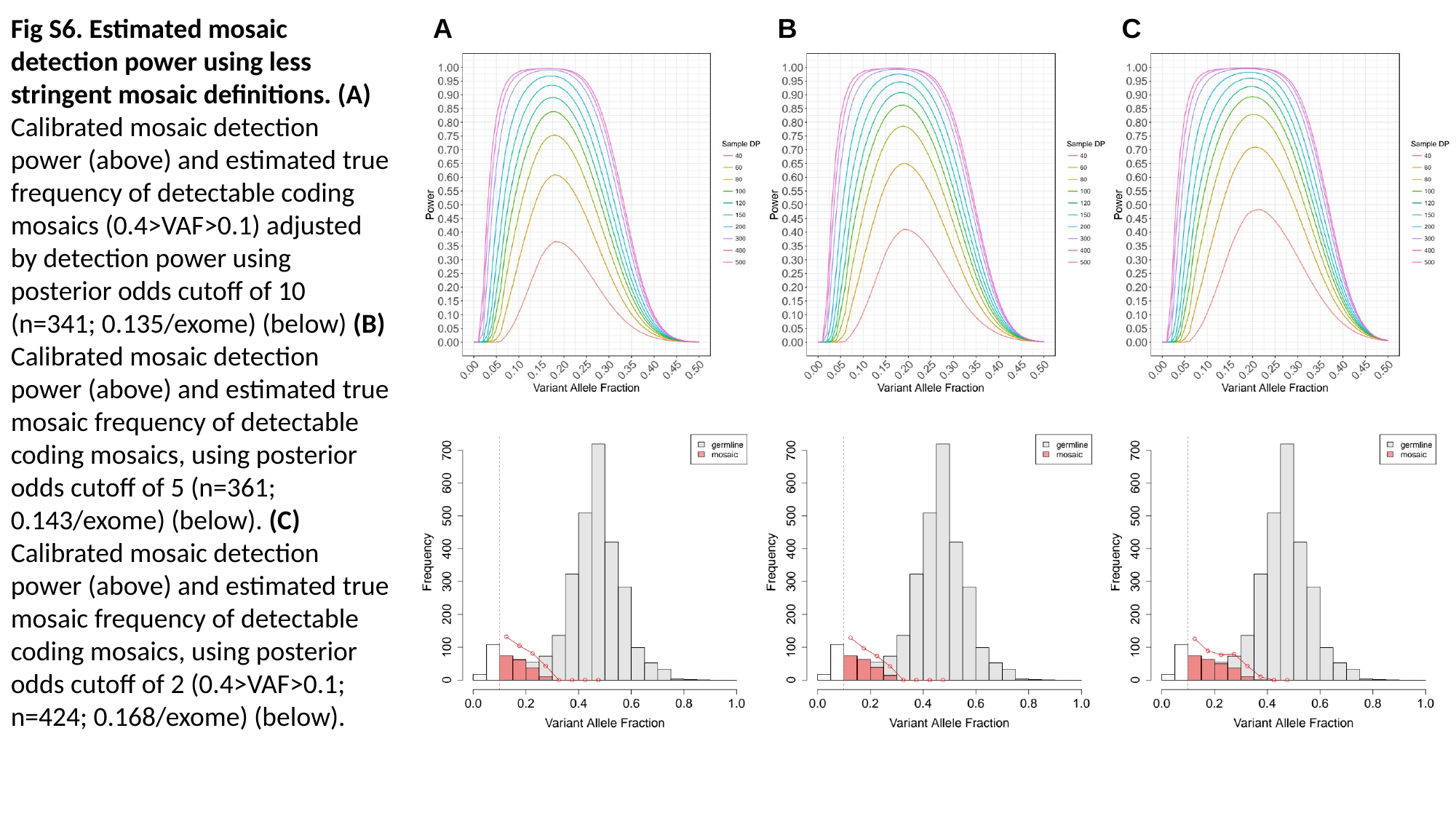

Fig S6. Estimated mosaic detection power using less stringent mosaic definitions. (A) Calibrated mosaic detection power (above) and estimated true frequency of detectable coding mosaics (0.4>VAF>0.1) adjusted by detection power using posterior odds cutoff of 10 (n=341; 0.135/exome) (below) (B) Calibrated mosaic detection power (above) and estimated true mosaic frequency of detectable coding mosaics, using posterior odds cutoff of 5 (n=361; 0.143/exome) (below). (C) Calibrated mosaic detection power (above) and estimated true mosaic frequency of detectable coding mosaics, using posterior odds cutoff of 2 (0.4>VAF>0.1; n=424; 0.168/exome) (below).
A
B
C

## Slide 8
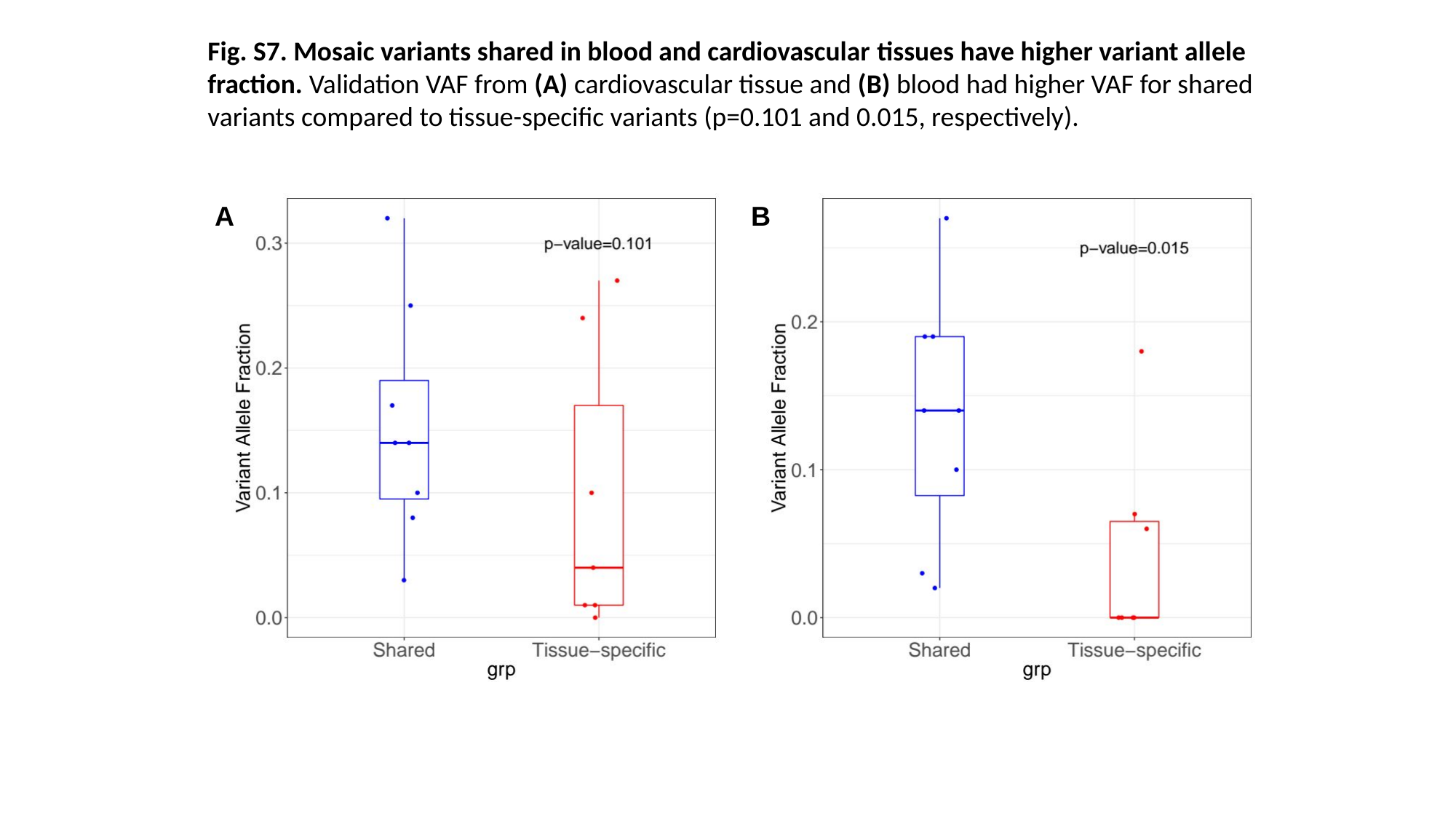

Fig. S7. Mosaic variants shared in blood and cardiovascular tissues have higher variant allele fraction. Validation VAF from (A) cardiovascular tissue and (B) blood had higher VAF for shared variants compared to tissue-specific variants (p=0.101 and 0.015, respectively).
A
B

## Slide 9
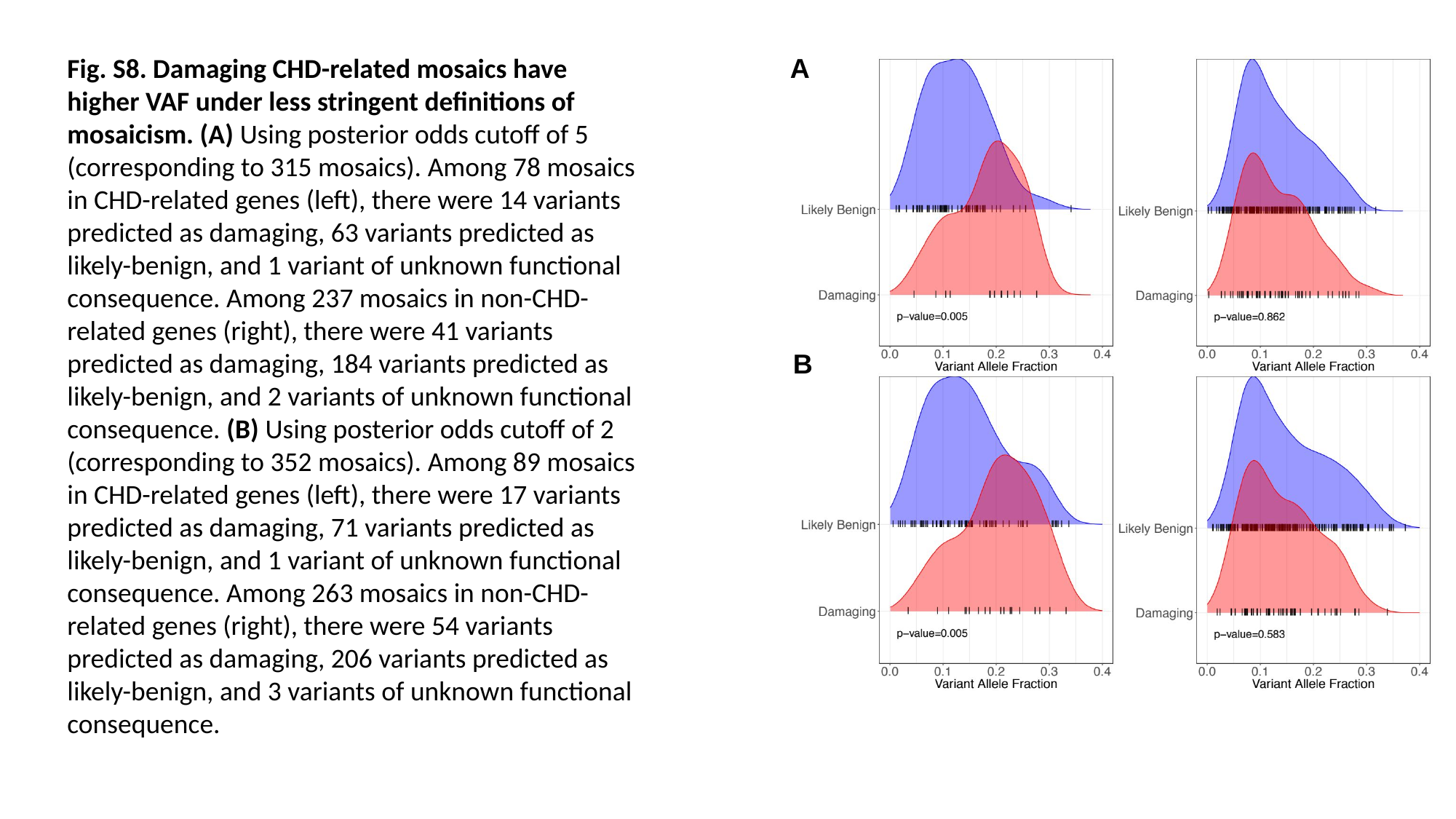

Fig. S8. Damaging CHD-related mosaics have higher VAF under less stringent definitions of mosaicism. (A) Using posterior odds cutoff of 5 (corresponding to 315 mosaics). Among 78 mosaics in CHD-related genes (left), there were 14 variants predicted as damaging, 63 variants predicted as likely-benign, and 1 variant of unknown functional consequence. Among 237 mosaics in non-CHD-related genes (right), there were 41 variants predicted as damaging, 184 variants predicted as likely-benign, and 2 variants of unknown functional consequence. (B) Using posterior odds cutoff of 2 (corresponding to 352 mosaics). Among 89 mosaics in CHD-related genes (left), there were 17 variants predicted as damaging, 71 variants predicted as likely-benign, and 1 variant of unknown functional consequence. Among 263 mosaics in non-CHD-related genes (right), there were 54 variants predicted as damaging, 206 variants predicted as likely-benign, and 3 variants of unknown functional consequence.
A
B

## Slide 10
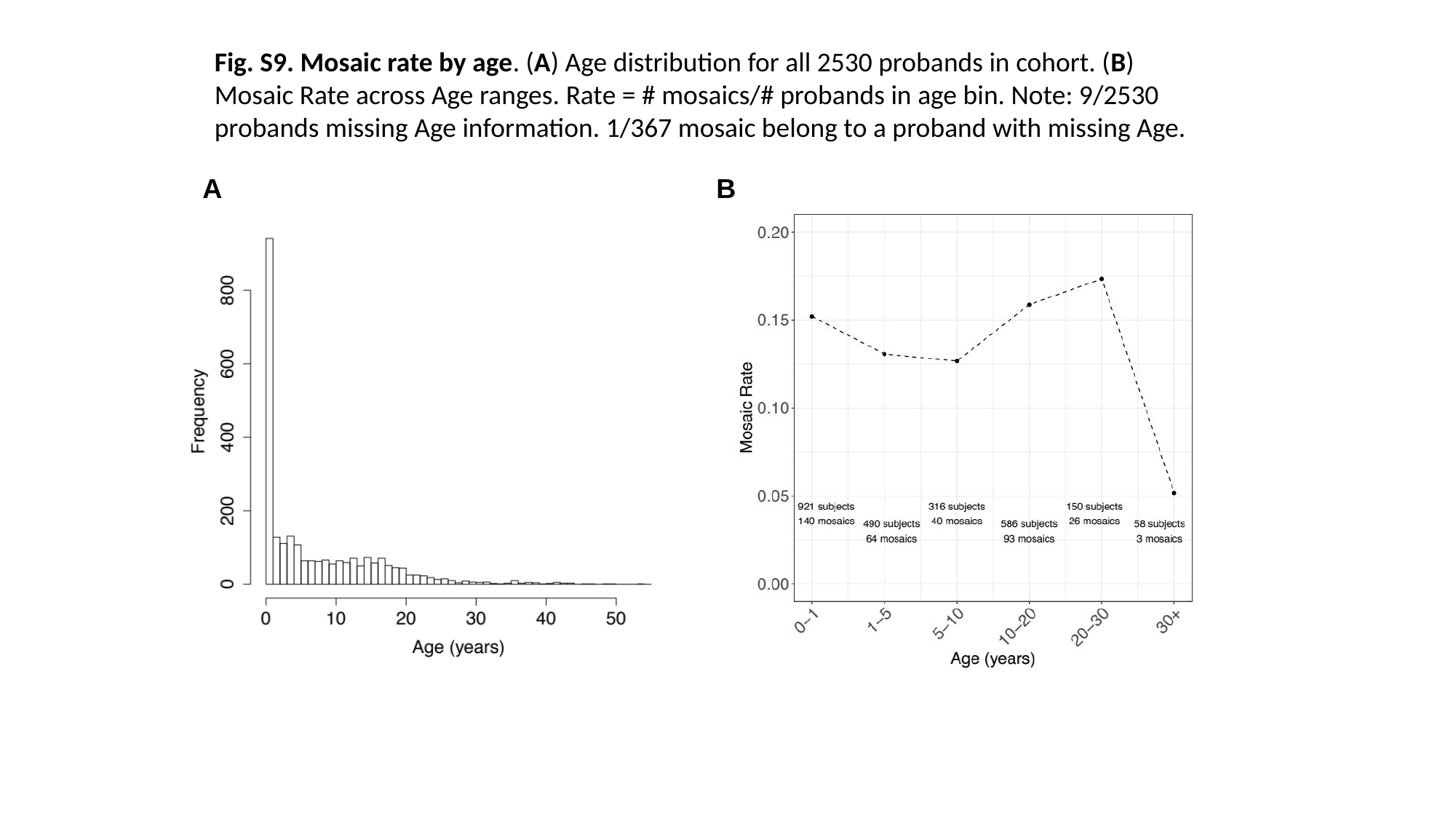

Fig. S9. Mosaic rate by age. (A) Age distribution for all 2530 probands in cohort. (B) Mosaic Rate across Age ranges. Rate = # mosaics/# probands in age bin. Note: 9/2530 probands missing Age information. 1/367 mosaic belong to a proband with missing Age.
A
B

## Slide 11
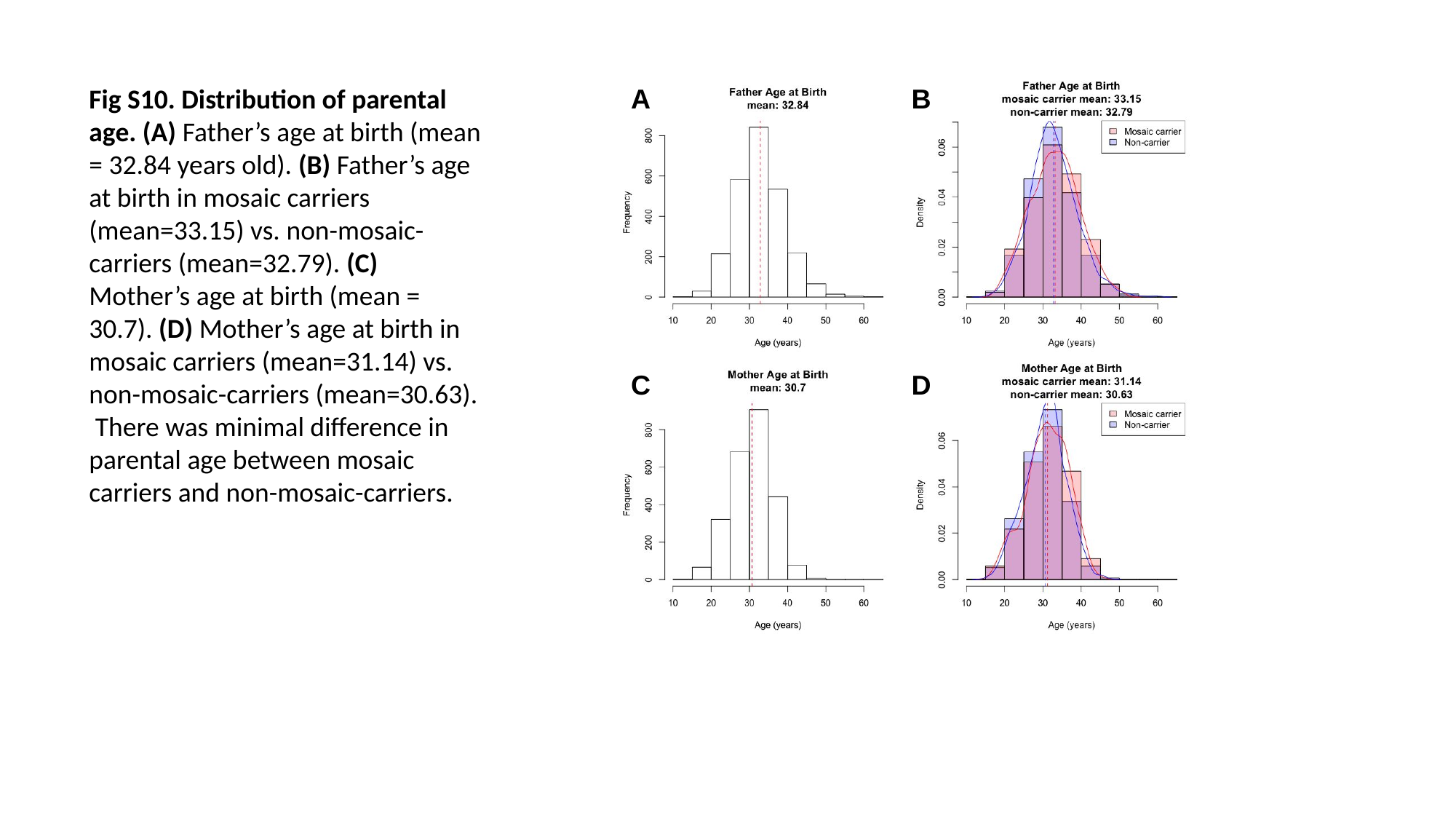

Fig S10. Distribution of parental age. (A) Father’s age at birth (mean = 32.84 years old). (B) Father’s age at birth in mosaic carriers (mean=33.15) vs. non-mosaic-carriers (mean=32.79). (C) Mother’s age at birth (mean = 30.7). (D) Mother’s age at birth in mosaic carriers (mean=31.14) vs. non-mosaic-carriers (mean=30.63). There was minimal difference in parental age between mosaic carriers and non-mosaic-carriers.
A
B
C
D

## Slide 12
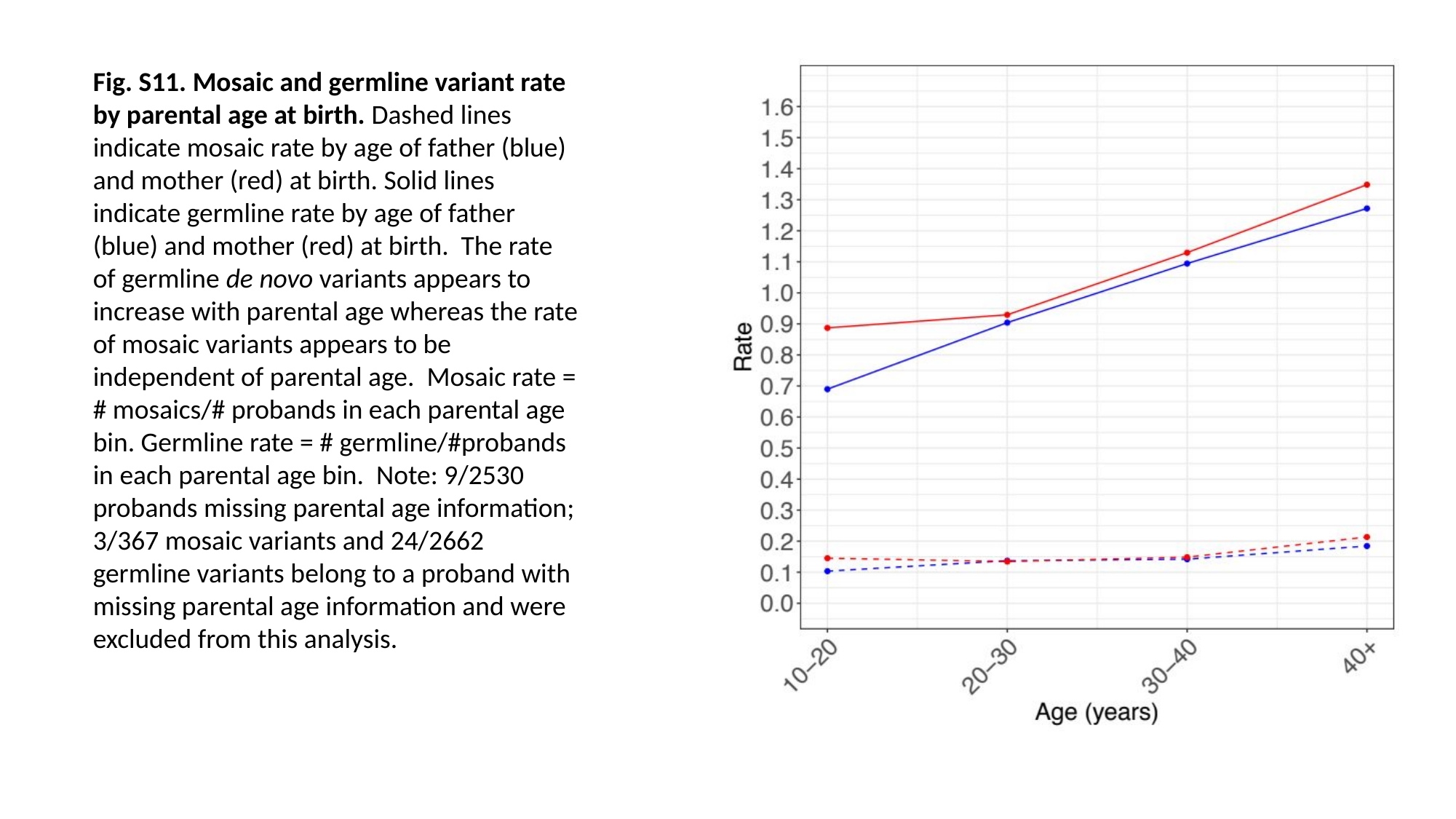

Fig. S11. Mosaic and germline variant rate by parental age at birth. Dashed lines indicate mosaic rate by age of father (blue) and mother (red) at birth. Solid lines indicate germline rate by age of father (blue) and mother (red) at birth. The rate of germline de novo variants appears to increase with parental age whereas the rate of mosaic variants appears to be independent of parental age. Mosaic rate = # mosaics/# probands in each parental age bin. Germline rate = # germline/#probands in each parental age bin. Note: 9/2530 probands missing parental age information; 3/367 mosaic variants and 24/2662 germline variants belong to a proband with missing parental age information and were excluded from this analysis.

## Slide 13
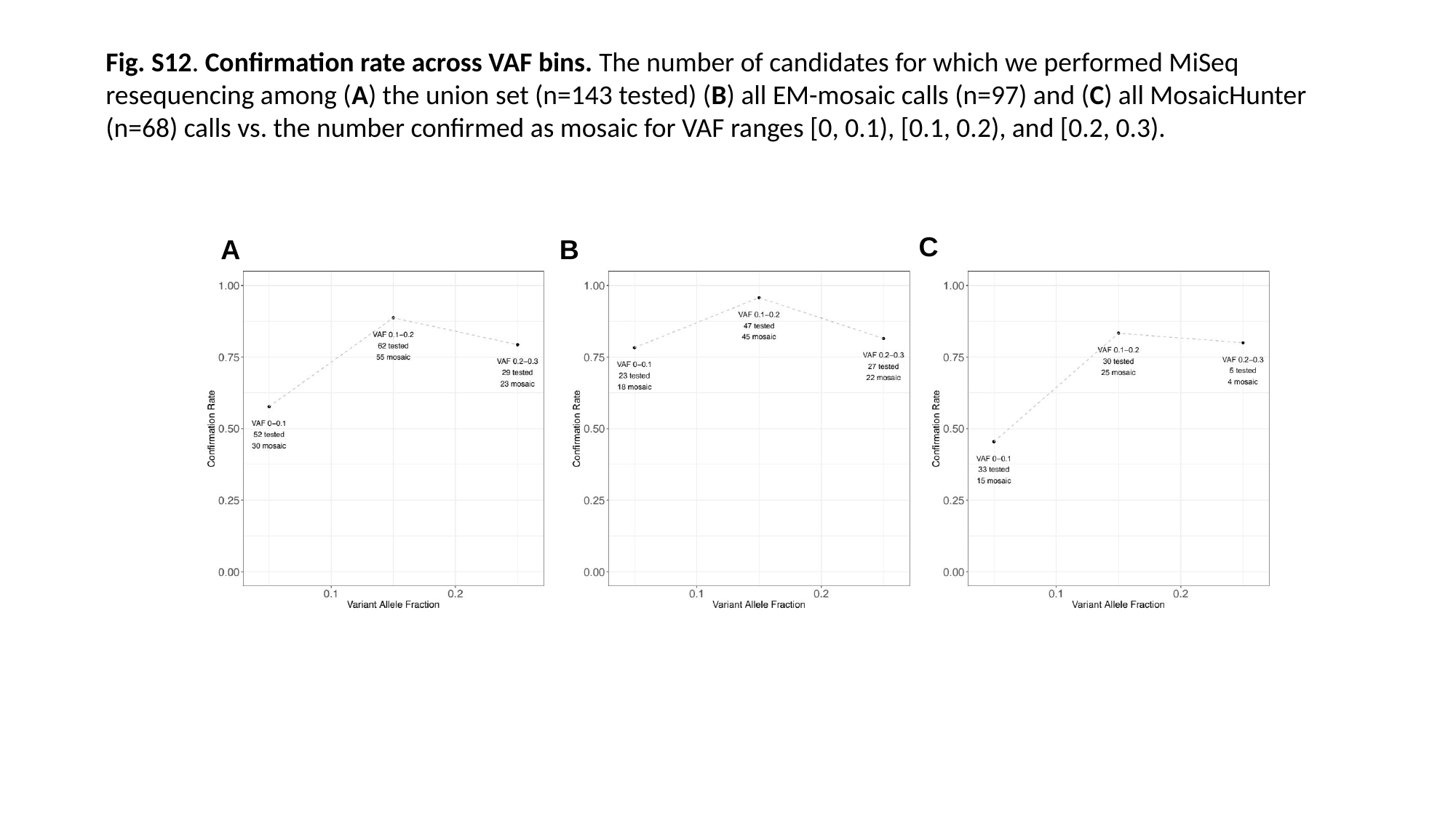

Fig. S12. Confirmation rate across VAF bins. The number of candidates for which we performed MiSeq resequencing among (A) the union set (n=143 tested) (B) all EM-mosaic calls (n=97) and (C) all MosaicHunter (n=68) calls vs. the number confirmed as mosaic for VAF ranges [0, 0.1), [0.1, 0.2), and [0.2, 0.3).
C
A
B

## Slide 14
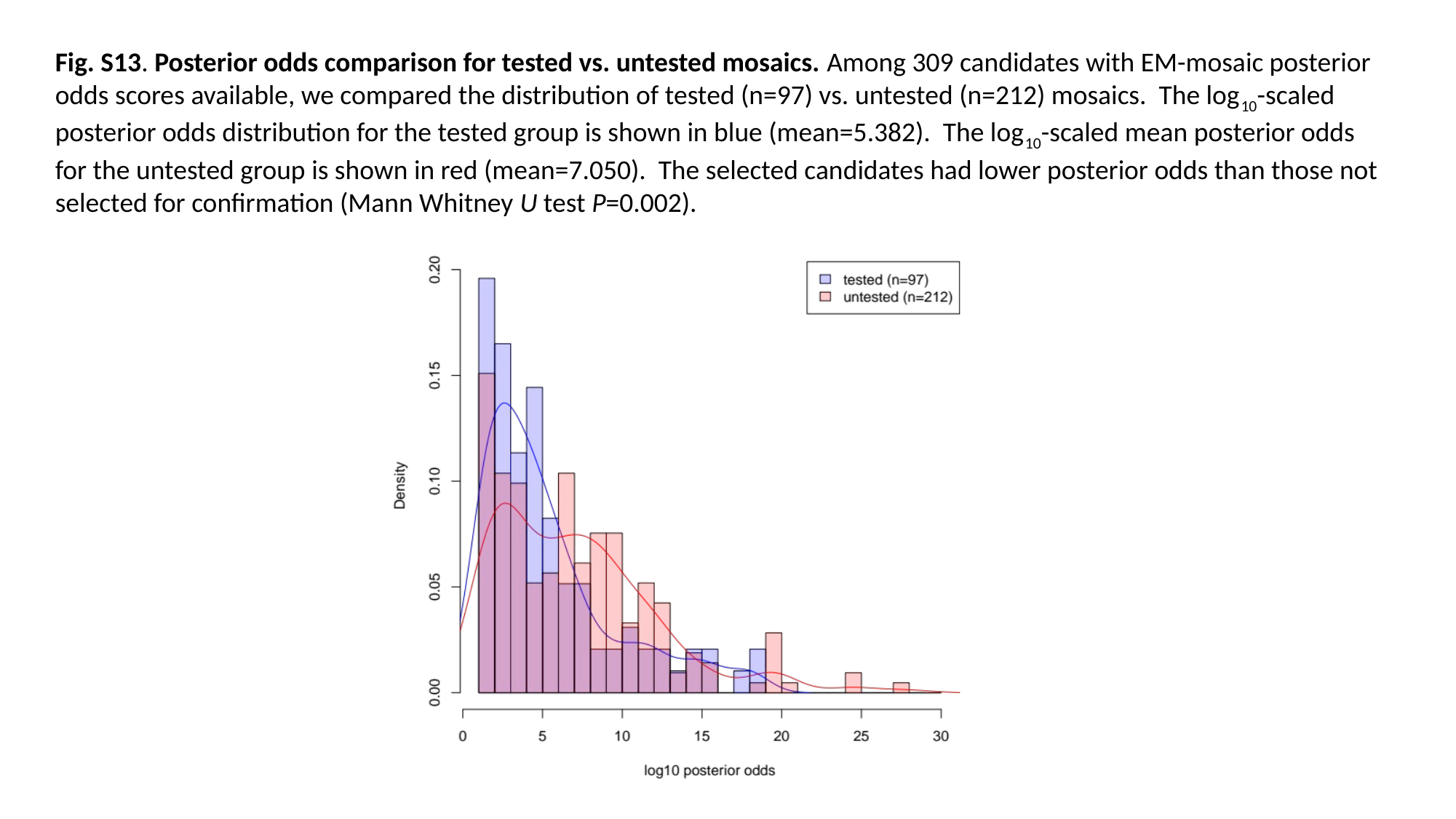

Fig. S13. Posterior odds comparison for tested vs. untested mosaics. Among 309 candidates with EM-mosaic posterior odds scores available, we compared the distribution of tested (n=97) vs. untested (n=212) mosaics. The log10-scaled posterior odds distribution for the tested group is shown in blue (mean=5.382). The log10-scaled mean posterior odds for the untested group is shown in red (mean=7.050). The selected candidates had lower posterior odds than those not selected for confirmation (Mann Whitney U test P=0.002).

## Slide 15
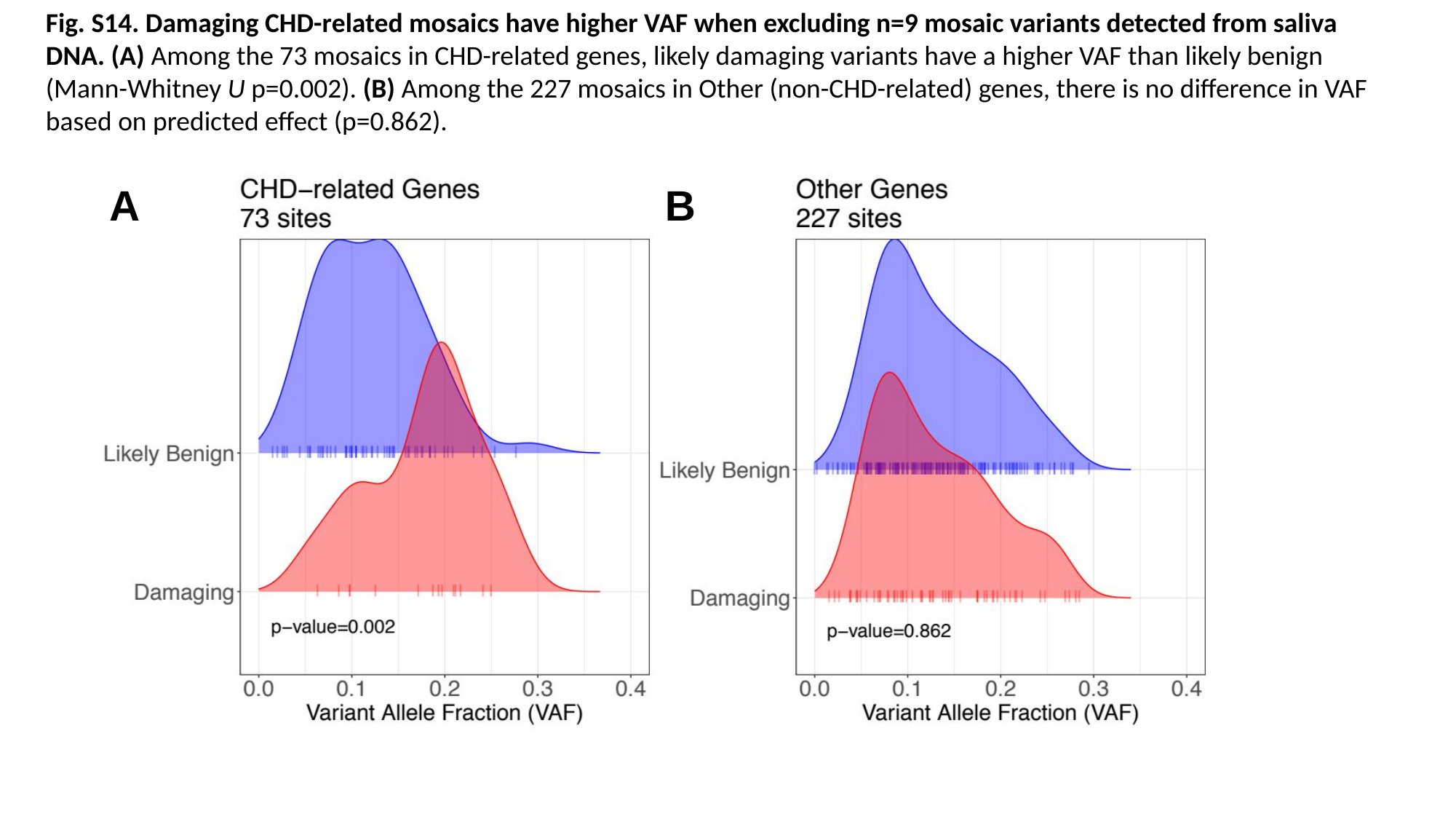

Fig. S14. Damaging CHD-related mosaics have higher VAF when excluding n=9 mosaic variants detected from saliva DNA. (A) Among the 73 mosaics in CHD-related genes, likely damaging variants have a higher VAF than likely benign (Mann-Whitney U p=0.002). (B) Among the 227 mosaics in Other (non-CHD-related) genes, there is no difference in VAF based on predicted effect (p=0.862).
A
B
