# Supplementary material for: EM-mosaic detects mosaic point mutations that contribute to congenital heart disease
Source: Genome Med. 2020 Apr 29;12:42. doi: 10.1186/s13073-020-00738-1 (PMC7189690; doi:10.1186/s13073-020-00738-1)
Supplement: Supplementary file 3 — Contains Supplemental Methods. [file 13073_2020_738_MOESM3_ESM.docx]

**Supplementary materials**

**Supplemental Methods**

*Union with Validated de novo SNVs from Jin et al. Nature Genetics 2017*

As part of the PCGC program, Jin et al. previously sequenced and processed a cohort of 2871 CHD probands – including 2530 parent-offspring trios used in this study – to investigate the contribution of rare inherited and *de novo* variants to CHD. They called a total of 2992 proband *de novo* variants, including 2872 SNVs and 118 indels, and Sanger confirmed a subset of the most likely-disease causing variants. Since we processed the same proband-parent trios using different variant calling pipelines, we combined the results of our two approaches to provide a more complete input *de novo* call set for mosaic variant detection.

We first processed our SAMtools *de novo* calls using our upstream filters (*n*=2396 sites passing all filters). We then applied the same upstream filters to the published dnSNVs from Jin et al. (*n*=2650 sites passing all filters) before finally taking the union of these two call sets (*n*=3192). There were 1814 sites in the intersection, with 836 sites unique to the Jin et al. calls and 542 sites unique to our SAMtools calls. After preprocessing, outlier removal, and FDR-based minimum N_alt_ filtering, the remaining 2971 dnSNVs were used as input to our mosaic detection model.

*Mutation Spectrum Analysis*

We compared the mutation spectrum – the frequencies of all possible base changes – of our predicted mosaic candidates against the spectrum of our predicted germline heterozygous variants. Under the assumption that that post-zygotic events occur randomly (i.e. due to errors in DNA replication rather than a specific biological process), the mosaic mutation spectrum should not differ significantly from the germline mutation spectrum. We used Pearson’s Chi-square Test to test for a difference in frequencies across all base changes between our predicted sets of variants. We interpreted large qualitative differences in base change frequencies as evidence of technical artifacts and rejection of the Chi-square null as evidence of systemic issues in our pipeline.

*Mosaic Detection Power Given Sample Average Coverage*

To model statistical power in the context of mosaic variant detection, we considered two conditional probabilities: (i) the probability of detecting a mosaic event (i.e. the probability of a variant’s posterior odds exceeding a threshold) given site depth *DP_site_*, VAF, and overdispersion parameter *θ* and (ii) the probability of observing site depth *DP_site_*, given sample-wide average coverage *DP_sample_*.

(i) Pr(detect mosaic | *DP_site_*, VAF, *θ*) was calculated by first identifying the VAF range (and by extension, the range of *N_alt_*) over which posterior odds > cutoff, then by integrating the beta-binomial probability mass function over this range, with considerations for the probability of strand bias (P(strand bias | *DP_site_*) ~ Binomial(*N_alt_*, *DP_site_*, *p*=0.5)).

(ii) Pr(*DP_site_* | *DP_sample_*) follows an overdispersed poisson distribution that we approximated using a negative binomial model with overdispersion parameter *θ* {Sampson 2011}. For each *DP_sample_* value, we calculated a vector of weights corresponding to Pr(*DP_site_* | *DP_sample_*) for *DP_site_* values in the range (1, 1500).

Finally, we took the sum of the detection probabilities described in (i) multiplied by the weights described in (ii) to determine the probability of detecting a mosaic variant given a sample average coverage value – Pr(detect mosaic | *DP_sample_*). Our estimated detection power curves for a range of sample average coverage values typical of exome-sequencing studies are shown in **(Figure 2C).** Our CHD cohort was sequenced to sample average depth of 60x, with prior mosaic fraction=0.121 and estimated *θ*=116*.*

To estimate the true rate of mosaicism per exome given sample average coverage, we first split our set of predicted mosaics into VAF bins of size 0.05. For each bin above VAF 0.1, we multiplied the number of mosaics by the inverse of the detection power for that given VAF bin to estimate the true count of mosaic variants in that VAF range, assuming full detection power. Since EM-mosaic is underpowered to detect mosaics with VAF < 0.1 in the blood and since this range is enriched for technical artifacts that potentially affect our counts, we did not apply this scaling procedure to these bins to avoid over-inflating our adjusted mosaic rate estimate **(Figure 6A, below)**.

*Filtering of MosaicHunter Candidate Variants*

MosaicHunter was used to identify candidate mosaic variants from blood exome-sequencing trio data using default settings {Huang 2014}. Filtering of original MosaicHunter candidate variants excluded, in order, any variant present in ExAC (46634), G to T mutations with fewer than N_alt_<10 oxidative indicating DNA damage {Costello 2013} (3995), non-uniquely called sites (4719), germline SNVs previously called by GATK HaplotypeCaller (591), probands with >20 mosaic variants (1490 in 10 probands), mosaic log posterior likelihood ratio <10 (940), variants with >2 parental alternative allele reads (244), variants with gnomAD population frequency > 1e-4 or located in MUC or HLA genes (40).

*Filtering of cardiovascular* *tissue Candidate Variants*

We used the MosaicHunter pipeline in trio mode to identify candidate variants in WES data from 70 cardiovascular tissue samples (belonging to 66 unique probands). From the list of variants initially reported by the pipeline using default settings, we applied the same filtration steps listed for MosaicHunter candidate variants in blood samples with the exception of the removal of G to T mutations with fewer than 10 alternative allele reads and the mosaic log posterior likelihood ratio <10. Finally, we removed variants that were identified in either parent or had a total read depth <10 in either parent.

*Clinical interpretation of mosaic variants – limitations*

We note that conventional clinical interpretation of mosaic mutations is challenging for several reasons: (i) it is unclear in which tissues each mosaic mutation is expressed (ii) several study participants were very young at time of clinical assessment and many classical disease features may not yet have developed or been noted, and (iii) the absence of additional clinical features does not necessarily rule out a mosaic mutation as being for the cause of the CHD. For the purposes of this study, we selected these mosaic mutations on the basis of predicted pathogenicity and detection in genes involved in biological processes relevant to CHD or developmental disorders

**Reference**

Sampson J, Jacobs K, Yeager M, Chanock S, Chatterjee N. Efficient study design for next generation sequencing. Genet Epidemiol. 2011;35(4):n/a. https://doi.org/10.1002/gepi.20575.
